# An integrated computational-experimental approach reveals *Yersinia pestis* genes essential across a narrow or a broad range of environmental conditions

**DOI:** 10.1186/s12866-017-1073-8

**Published:** 2017-07-21

**Authors:** Nicola J. Senior, Kalesh Sasidharan, Richard J. Saint, Andrew E. Scott, Mitali Sarkar-Tyson, Philip M. Ireland, Helen L Bullifent, Z. Rong Yang, Karen Moore, Petra C. F. Oyston, Timothy P. Atkins, Helen S. Atkins, Orkun S. Soyer, Richard W. Titball

**Affiliations:** 1 College of Life and Environmental Sciences, University of Exeter, Exeter, EX4 4SB UK; 2 School of Life Sciences, University of Warwick, Coventry, CV4 7AL UK; 3 Defence Science Technology Laboratory, Porton Down, Salisbury, SP4 OJQ UK; 4 Marshall Centre for Infectious Disease Research and Training, School of Pathology and Laboratory Medicine, University of Western Australia, Perth, WA 6009 Australia

**Keywords:** *Yersinia pestis*, Plague, TRADIS, Transposon, Essential genes, Metabolic model

## Abstract

**Background:**

The World Health Organization has categorized plague as a re-emerging disease and the potential for *Yersinia pestis* to also be used as a bioweapon makes the identification of new drug targets against this pathogen a priority. Environmental temperature is a key signal which regulates virulence of the bacterium. The bacterium normally grows outside the human host at 28 °C. Therefore, understanding the mechanisms that the bacterium used to adapt to a mammalian host at 37 °C is central to the development of vaccines or drugs for the prevention or treatment of human disease.

**Results:**

Using a library of over 1 million *Y. pestis* CO92 random mutants and transposon-directed insertion site sequencing, we identified 530 essential genes when the bacteria were cultured at 28 °C. When the library of mutants was subsequently cultured at 37 °C we identified 19 genes that were essential at 37 °C but not at 28 °C, including genes which encode proteins that play a role in enabling functioning of the type III secretion and in DNA replication and maintenance. Using genome-scale metabolic network reconstruction we showed that growth conditions profoundly influence the physiology of the bacterium, and by combining computational and experimental approaches we were able to identify 54 genes that are essential under a broad range of conditions.

**Conclusions:**

Using an integrated computational-experimental approach we identify genes which are required for growth at 37 °C and under a broad range of environments may be the best targets for the development of new interventions to prevent or treat plague in humans.

**Electronic supplementary material:**

The online version of this article (doi:10.1186/s12866-017-1073-8) contains supplementary material, which is available to authorized users.

## Background

*Yersinia pestis* is a Gram-negative bacterium and the causative agent of plague [1]. Various schemes have been proposed to sub-type *Y. pestis*, and the most frequently used places strains into one of three biovars (Antiqua, Mediaevalis or Orientalis) on the basis of the differential ability of isolates to ferment glycerol and reduce nitrate [2]. Of these, biovar Orientalis strains are associated with the most recent outbreaks of disease. Plague remains a threat in many parts of the world, particularly Africa [1], and the World Health Organization has categorized plague as a re-emerging disease. There is currently no licensed vaccine against plague in the West, while a multi-drug resistant strain has emerged in Madagascar [3]. These findings and the potential of *Y. pestis* to be used as a bioweapon [4] make the findings of this study highly relevant towards identifying new drug targets against this pathogen.

*Y. pestis* can infect a wide range of mammalian hosts [1, 5]. Although it can be transmitted in aerosols to generate primary pneumonic plague or via ingestion, most natural cases arise following the bite of an infected flea [1]. Plague can survive and replicate in the digestive tract of the flea and as the infected flea attempts to feed, bacteria are regurgitated into the bite site and the mammalian host is infected. Therefore, the bacterium can survive in two distinct environments and a key environmental stimulus affecting gene expression in these niches is temperature. In the flea, typically at 22–30 °C genes such as those responsible for extracellular matrix and biofilm formation and for the murine toxin play key roles. [6]. In the mammalian host, virulence factors are upregulated at 37 °C, including the F1 antigen capsule and the plasmid pCD1-encoded type III secretion system [7].

Next-generation sequencing and transposon mutagenesis are powerful technologies for the genome-wide identification of gene functions in bacterial pathogens [8]. When combined, they allow the precise mapping of the DNA sequence spanning the junction between the transposon and the recipient DNA [8]. Consequently, millions of mutants can be simultaneously and individually monitored for their frequency within a population. By exposing the population to a specific stress, mutants that are disadvantaged can be revealed [9]. Consequently it has been possible to identify genes that are necessary for growth [8]. These genes are “essential” because their inactivation results in a fitness disadvantage, within an otherwise wildtype population. Essential genes or their products can be targets for novel therapeutics [10]. Depending on the transposon used and the sequencing methodology employed, the technology has been called INSeq (insertion sequencing), Tn-seq (transposon sequencing), TraDIS (transposon-directed insertion site sequencing) or HITS (high-throughput insertion tracking by deep sequencing) and these different methods have recently been reviewed [11].

In conjunction with this powerful experimental approach, it is possible to apply computational tools to predict essential genes under different conditions and for a wide range of pathogens. This integrative approach can provide mechanistic explanations for experimentally identified essential genes, such as identifying conditions which modify gene essentiality. To this end several studies explored the possibility of identifying essential genes from genome-scale, stoichiometric metabolic networks through the application of flux balance analysis [12, 13]. This approach allows prediction of biomass production under a defined media composition and subsequent interrogation of the effects of in silico deletion of genes on growth, i.e. gene essentiality. While such computational prediction of gene essentiality is attractive, to date, there are not many systematic studies that perform computational predictions under different media conditions and compare these predictions on gene essentiality with corresponding experimental studies in the same organism.

Previously, we developed a novel algorithm for the experimental identification of essential genes in *Y. pestis* CO92 grown at 28 °C and identified 548 essential genes [14]. Here, we apply an integrated computational-experimental approach to investigate gene essentiality in *Y. pestis* grown at 28 °C and 37 °C. The application of experimental mutagenesis and metabolic modelling in this study highlights the presence of environmental condition-specific, as well as “core” essential genes. The latter group of genes are predicted to be required for growth under a broad range of environments and represent preferential targets for the development of new interventions to control disease.

## Methods

### Generation of mutants

*Y. pestis* strain CO92 was originally isolated from a fatal human case of primary pneumonic plague contracted from an infected cat and has been genome sequenced [15]. The bacteria were cultured in blood agar base (BAB) broth or BAB agar supplemented with hemin (0.025% *w*/*v*) at 28 °C. When required, media was supplemented with kanamycin (25 μg ml^−1^), trimethoprim (100 μg ml^−1^), chloramphenicol (25 μg ml^−1^). L-rhamnose (0.02%) or L-glucose (0.1%). A library of over 1 million mutants was constructed in *Y. pestis* CO92 by random mutagenesis using the EZ-Tn5 < kan-2 > Tnp transposome kit (Epicentre) according to the manufacturer’s instructions. *Y. pestis* was cultured in broth at 28 °C and was made electro-competent by sequential washes in 10% glycerol. Parameters defined previously for the electroporation of *Y. pestis* to high efficiency were applied [16]. After electroporation, cells recovered for 2 h in BAB broth prior to plating. Mutants were washed from the plates and pooled into batches of approximately 2 × 10^5^ colonies before combining to create the final transposon library.

### Sequencing of mutants

Transposon libraries were cultured in BAB broth at 28 °C or 37 °C overnight and genomic DNA extracted using the Gentra Puregene kit (Qiagen). The gDNA was fragmented to <500 base pairs (bp) using 2 × 15 min cycles at 4 °C in a BioRuptor sonicator (medium intensity, 30s on/90s off). A NEBNext DNA library preparation for Illumina kit (NEB) was used according to the manufacturer’s instructions, to end repair, A-tail and ligate adapters to the fragments. The adapters used were Ind_Ad-T and Ind_Ad-B (Additional file 1: Table S1), which were annealed prior to use. Parallel polymerase chain reaction (PCR) samples were set up with 10 μl JumpStart 10× buffer, 6 μl MgCl_2_, 2 μl 10 mM nucleoside triphosphates (dNTPs), 0.6 μl 100 μM PE_PCR_V3.3 primer, 0.6 μl 100 μM Yp_EzTn_PCR primer, 1 μl JumpStart Taq DNA polymerase and 28.8 μl nuclease-free water per reaction. Primer sequences are listed in Additional file 2: Table S2. The reactions were amplified at 94 °C for 2 min, (94 °C for 30 s, 60 °C for 20 s, 72 °C for 30 s) for 22 cycles, 72 °C for 10 min, then held at 12 °C. PCR products were pooled and ethanol precipitated before being size selected on a 2% (*w*/*v*) agarose tris-borate-EDTA (TBE) gel. Agarose blocks corresponding to 350–500 bp were excised, and the DNA extracted using a Qiagen MinElute Gel Extraction kit as per the manufacturer’s instructions. The DNA was quantified by qPCR and on an Agilent BioAnalyzer before being submitted for sequencing as 100 bp single end reads on an Illumina HiSeq 2500 standard model. In total, there were 57.2 million raw sequencing reads. 45.8 million (80.1%) were transposon sequences. Nine million (19.7%) transposon sequences were mapped to the genome (AL59084). This sequence data has been submitted to the NCBI Sequence Read Archive (SRA) database under accession numbers GSE100226, GSM2674959, GSM2674960, GSM2674961, GSM2674962, GSM2674963, GSM2674964 and GSM2674965.

### Data processing of transposon insertion data

Data was processed using an algorithm that we have previously devised [14] and which predicts gene essentiality based on the number and location of transposon insertions within each gene. Genes that lacked any transposon insertions were termed Type I essential genes. The remaining genes were then subjected to noise trimming using a tight cluster approach, and those classed as essential by this were termed Type II essential genes. Finally, genes had their individual mutation features calculated and our algorithm used to identify Type III essential genes [14].

### Metabolic model for *Y. pestis* CO92

The previously reported metabolic model for *Y. pestis* CO92 [17] was downloaded and used without any alterations, except for the setting of exchange reaction bounds for simulation of different environments (see below).

### Functional gene analysis

The latest Cluster of Orthologous Groups (COGs) information was downloaded from ftp://ftp.ncbi.nih.gov/pub/COG/COG2014/data on June 27 2015. The functional category of each gene in the *Y. pestis* CO92 model was then identified from the COGs tables. Other relevant information, such as gene ID and GI number were obtained using NCBI E-utilities (http://eutils.ncbi.nlm.nih.gov).

### Metabolic gene essentiality analysis under different environments

In this analysis, the metabolic model is simulated under a specific environment, which is set by constraining the exchange fluxes to specific values (exchange reactions manage the import and export of metabolites between the cell and the environment/medium). Within the specified environment, flux through each reaction in the model was disabled one at a time and the resulting model (corresponding to a single gene knockout mutant) was optimised for biomass objective function (BOF) as before [18]. If the altered model did not result in any feasible optima (if flux through the BOF was less than 1% of the original growth rate; i.e. < 0.0028), then the gene corresponding to the disabled reaction was considered as essential under that environment.

The BCS media is simulated in the model using the 21 exchange reactions involving metabolites found in that media, however, the original model has a total of 281 exchange reactions. That means, if we were to try all combinations of the exchange reactions to generate possible environments, there would be 2^281^ different environments (media compositions). This number is too large to analyse in a tractable manner under reasonable computational resources and time. Therefore, we have used the following two approaches to sample this possible environment space to obtain a set of feasible number of diverse media compositions: (a) generate all the combinations of the 17 out of 21 exchange reactions used in the BCS medium (in this approach four exchange reactions were always supplied because they are found to be essential for growth in BCS medium; L-methionine, O_2_, L-phenylalanine and phosphate), (b) generate over 2 million random combinations of all the exchange reactions (L-phenylalanine was supplied in all random media as it is found to be an essential metabolite for growth in all the media).

By setting a theoretical, “maximal environment” that contained all substrates that the model can take up (i.e. the lower-bounds of all exchange reactions are set to −1000), we identified a set of “super” essential genes. The BOF feasibility cut-off used in this analysis was 1% of the growth rate under the maximal environment; i.e. <1.2244.

## Results and discussion

### Experimental identification of *Y. pestis* CO92 essential genes

We previously reported the construction of a library of over 1 million mutants in *Y. pestis* CO92 by random mutagenesis using the EZ-Tn5 < kan-2 > Tnp transposon [14]. Genes that were essential for growth of *Y. pestis* at 28 °C were identified using TraDIS. We processed data from three biological replicates, and identified 530 genes which were essential when the bacteria were cultured at 28 °C. In this study we grew the library at 37 °C and identified genes essential at this temperature.

**Fig. 1:**
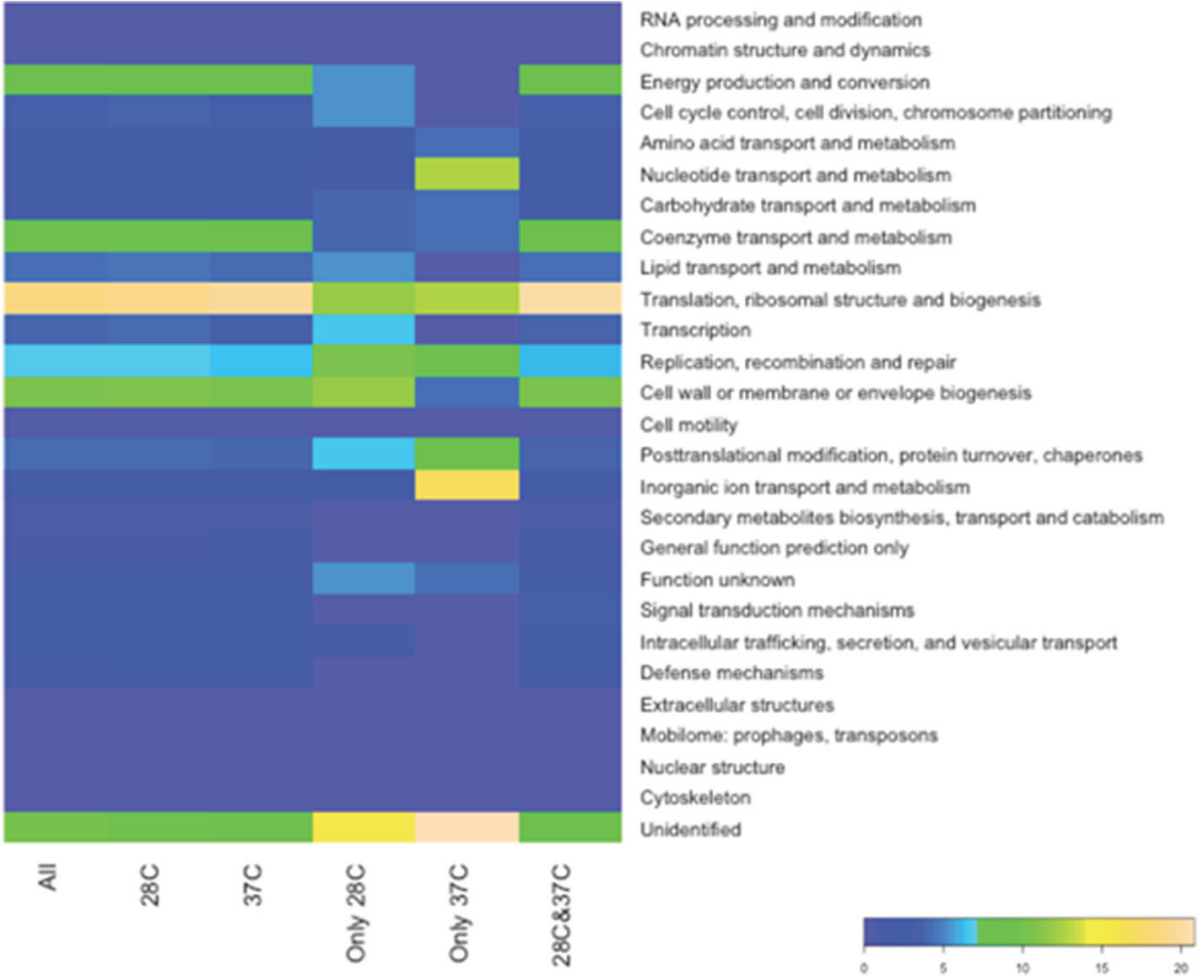
Analysis of the experimentally identified *Y. pestis* CO92 essential genes. A heat-map showing the percentage of COGs in different groups of essential genes (x axis); “All”, all essential genes; “28C”, genes that are essential at 28 °C; “37C”, genes that are essential at 37 °C; “Only 28C”, genes that are essential only at 28 °C; “Only 37C”, genes that are essential only at 37 °C; “28C and 37C”, essential genes common at both temperatures

In order to study the functions of the experimentally identified essential genes we used COG data information on *Y. pestis* CO92 proteins. The most frequently represented groups, at 28 °C or 37 °C, were those including “translation, ribosomal structure and biogenesis” and “cell wall or membrane or envelope biogenesis” proteins. In the combined 28 °C and 37 °C datasets these groups were 18.7% and 10.5% respectively of the total (Fig. 1). However both the 28 °C and 37 °C essential gene lists included “unidentified” genes (Fig. 1).

The “replication, recombination and repair” (10.5%) cluster was prominent among the genes that were essential only at 28 °C, whereas “Inorganic ion transport and metabolism” (16.7%), “translation, ribosomal structure and biogenesis” (12.5%) and “nucleotide transport and metabolism” (12.5%) clusters were dominant among the genes that are essential only at 37 °C.

### Genes essential at 37 °C, but not at 28 °C, may play roles in virulence

We identified 19 genes which were essential at 37 °C but not at 28 °C (Table 1). Several of these are likely to play a role in enabling the type III system, which is expressed at 37 °C but not at 28 °C [19]. The type III system is a major virulence determinant in *Y. pestis* and its expression can result in the suppression of bacterial growth [19]. Previously a *pspC* mutant of *Yersinia enterocolitica* has been shown to be attenuated in mice, and defective in growth in vitro when the type III system was induced (16). This is believed to reflect the ability of the Psp response to protect the cell envelope after induction of the type III system (17). Furthermore a *pspB* mutant showed sensitivity to overexpression of the YsaC secretin component of the type III system. It is believed that this is because misfolded secretin is lethal in the absence of the Psp response [20]. The *trkA* gene encodes a peripheral membrane protein and is a key component of the Trk low affinity K+ transport system in Gram negative bacteria [21]. In *S. enterica*, reduced intracellular growth and virulence has been linked to the decreased secretion of SPI1 type III system effectors by a *trkA* mutant [22]. TrkA has also been implicated in sensitivity to overexpression of the YsaC secretin in *Y. enterocolitica* [20], mirroring the phenotype of *pspB* mutants.

**Table 1:** Genes experimentally identified to be essential at 37 °C but not 28 °C

Gene number	Gene name	Gene product
YPO0239	*trkA*	potassium transporter peripheral membrane component
YPO0331	*ypo0331*	hypothetical protein
YPO0492	*ksgA*	dimethyladenosine transferase
YPO0892	*xerD*	site-specific tyrosine recombinase
YPO1020	*recB*	exonuclease v subunit beta
YPO1101	*smpB*	SsrA-binding protein
YPO1102	*ypo1102*	hypothetical protein
YPO1213	*nrdB*	ribonucleotide-diphosphate reductase subunit beta
YPO1391	*cmk*	cytidylate kinase
YPO2159	*ypo2159*	hypothetical protein
YPO2327	*ypo2327*	lipoprotein
YPO2350	*pspB*	phage shock protein B
YPO2883	*ndk*	nucleoside diphosphate kinase
YPO2894	*iscA*	iron-sulfur cluster assembly protein
YPO2907	*glyA*	serine hydroxymethyltransferase
YPO3173	*thii*	thiamine biosynthesis protein ThiI
YPO3696	*treC*	trehalose-6-phosphate hydrolase
YPO3847	*cyaY*	frataxin-like protein
YPO3864	*nfrC/wecB*	UDP-N-acetylglucosamine 2-epimerase

The *smpB* gene product might also play a role in the *Yersinia* type III system. SmpB interacts with transfer-messenger RNA (tmRNA) and along with SsrA they mediate ribosome recycling in the event of stalling on mRNA during translation [23]. In *Yersinia pseudotuberculosis* and *Y. pestis smpB*-*ssrA* mutants show reduced secretion of type III effectors at 37 °C and a *Y. pestis smpB*-*ssrA* mutant is markedly attenuated in mice [24].

Another group of genes that were essential at 37 °C but not at 28 °C (*nrdB*, *ndk*, *cmk*, *xerD* and *recB*) encode proteins associated with DNA replication and maintenance. NrdB is the β-subunit of ribonucleotide-diphosphate reductase, and plays a key role in the first step of synthesis of DNA precursors. In *Escherichia coli*, an *nrdB* mutant is temperature sensitive, which is believed to reflect the increased deoxyribonucleotide demand associated with increased growth rates at higher temperatures [25]. The *ndk* gene encodes a nucleoside diphosphate kinase, which plays a key role in the synthesis of nucleoside triphosphates such as GTP. Consequently, it is not surprising that the gene plays a role in virulence of pathogens such as *Mycobacterium tuberculosis* [26] and *Pseudomonas aeruginosa* [27] and is upregulated during *Salmonella enterica* infections [28]. In addition studies with *M. tuberculosis* show that *ndk* plays a role in phagosome maturation, promoting growth of the bacteria [26]. Thus in this case it appears to have an effector-like function. In some species, such as *S. enterica*, mutation of *ndk* imparts a cold sensitive phenotype [29]. Cytidine monophosphate kinase (Cmk) plays a crucial role in the recycling of nucleoside precursors to fuel DNA synthesis, acting alongside NdrB and a *cmk* mutant of *Y. pseudotuberculosis* is attenuated in mice [30]. Cmk mutants of other pathogens have been shown to have growth defects, and in some cases these are temperature dependent [31, 32]. The *xerD* gene is a component of the *xerCD* complex, which plays a role in the segregation of replicated chromosomal and plasmid DNA. This gene was essential for in vitro growth of *S. aureus* [33] and a *Brucella abortus xerD* mutant was attenuated in mice [34]. We also identified *recB* as essential at 37 °C but not 28 °C. RecB is a helicase and part of the RecBCD complex, which plays a key role in the repair of double stranded DNA breaks [35]. Although there is also redundancy in this pathway [35], the loss of RecBCD or RecB decreases the ability of bacteria to withstand stresses including elevated temperatures. Thus it is not surprising that *recB* mutants of pathogens such as *E. coli*, *S. enterica* [35] and *Helicobacter pylori* [36] are attenuated. Overall, it seems that these DNA replication and maintenance proteins are more essential at higher temperatures.

The remaining genes are predicted to play roles in stress responses (*cyaY* and *iscA*) [37, 38], the modification of adenosines in the small ribosome sub-unit (*ksgA*) [39], the generation of thiamine pyrophosphate, an essential co-factor for many enzymes (*thil*) [40], carbon metabolism (*glyA* and *treC*) and the biosynthesis of the enterobacterial common antigen (*nfrC/wecB*). Some of these genes have previously been shown to play a role in the virulence of *Y. pestis* (*wecB* [41]), *Y. pseudotuberculosis* (*ksgA* [42]) or other pathogens (*cyaY* [43], *glyA* [44–46], *treC* [47]).

The functions of four of these genes identified as essential at 37 °C but not at 28 °C (YPO0331, YPO1102, YPO2159 and YPO2327) could not be predicted on the basis of motif or homology matches, although YPO2327 is predicted to be a lipoprotein and is therefore likely to be surface located. These proteins should be a focus of future investigations to establish their possible roles in virulence. There was little overlap of our list of genes essential at 37 °C with previously reported data on genes upregulated at 37 °C in broth [48, 49] or in serum [50] or with the reported data on proteins with increased abundance in bacteria grown at 37 °C [51]. These findings highlight the importance of using several methodologies to obtain a robust picture of the molecular changes that occur in bacteria exposed to different conditions.

### Experimentally identified essential genes in *Y. pestis* CO92 overlap with, but differ from, those identified in *Y. pestis* KIM 1001

Palace et al. [52] previously reported the use of Tn-seq to probe the genome of *Y. pestis* KIM 1001 (biovar Mediaevalis) for elements contributing to fitness. Applying a Hidden Markov Method (HMM), Palace et al. classified genes as essential, growth-advantaged, growth-disadvantaged or non-essential at 37 °C. They identified 624 essential genes, including 19 located on the plasmids. We compared this list with the essential genes we identified in *Y. pestis* CO92 at 28 °C or 37 °C and found that 433 and 397 genes respectively were common to both studies. Of the 397 genes identified in both studies at 37 °C, 97% had homologues already identified in the Database of Essential Genes (DEG) (Additional file 3: Table S3). The differences in the two experimental lists of essential genes might reflect differences in the biology of these two strains of *Y. pestis*. *Y. pestis* strains CO92 and KIM10 share 95% of their sequence with each other, but a comparison of the genome sequences reveals numerous re-arrangements [53]. It is also possible that the differences reflect differences in the experimental methodologies used to identify essential genes. The Himar transposon system used by Palace et al. [52] in their Tn-Seq studies preferentially targets TA motifs, whereas the Tn5 system used in our study is more likely to insert into GC-rich sequences [54, 55]. Additionally, different methodologies have been used to analyse the data. We have used an algorithm we have developed, which takes into account both the positions and frequencies of mutations in the target gene, as well as the phenomenon of background noise from sequencing. In contrast, Palace et al. used an HMM based method [52]. Finally, the bacteria were cultured in different ways after transposon mutagenesis. In our study *Y. pestis* CO92 was cultured in BAB-hemin whereas Palace et al. used TB with zeocin [52]. This raises the possibility that identification of essential genes might be dependent on the growth media and conditions [56, 57].

Many of the genes which were essential in both KIM1001 and CO92 have previously been identified as playing a role in central metabolism, and the finding that their disruption results in an effect on growth is therefore not surprising. For example, 46 of the genes encoded ribosomal proteins, 28 genes involved in carbon metabolism and 4 TCA cycle genes were identified as playing a role in the growth of both KIM10 and CO92. More interesting are those genes encoding proteins which are not found in humans, or where the human homologue is structurally diverse from the bacterial protein. These bacterial proteins might be exploited as drug targets. For example genes encoding proteins in the type II and Tat export pathways were identified as essential in both strains and the Tat system is currently being investigated as a target for small molecule inhibitors [58]. Five genes involved in lipopolysaccharide biosynthesis were identified as essential and may also be good drug targets. Other genes essential in both KIM1001 and CO92 which encode gene products unique to bacteria, such as the FtsZ cell division protein, the twin arginine transport (Tat) system and the SurA chaperone, encode potential drug targets. For example, currently there is significant interest in developing inhibitors of FtsZ [59]. Our study also identified novel targets for antimicrobial drugs including the hypothetical proteins YPO3498, YPO3579, YPO3586 and YPO3665/6/7 which encode the rod shape-determining proteins MreB, MreC and MreD respectively. Collectively these findings highlight the potential to identify novel drug targets from essential genes.

Our data can also be used to identify mutations which result in reduced growth, and which could be exploited to generate live attenuated vaccines. Mutations in essential genes in both KIM1001 and CO92, including those encoding the catabolite repressor protein Crp [60, 61], components of the translational quality control system SmpB/SsrA [24], SpoT which is involved in the ppGpp synthesis [62] and Dam which is involved in DNA adenine methylation [63] have all been shown to be attenuated in mice and dosing with these mutants induces protective immunity.

### Computational analysis of a metabolic model of *Y. pestis* CO92 reveals environment-dependency of gene essentiality

To explore potential dependency of gene essentiality on growth conditions, and to further verify our experimental gene essentiality list from a metabolic stance, we utilised a genome-scale metabolic network reconstruction of *Y. pestis* CO92 [17]. This model includes 815 metabolic genes, out of which 226 were identified as essential from our experimental study reported here. Using flux balance analysis (FBA) [18], we interrogated this model for metabolic reaction fluxes that can support pathogen growth in a given growth medium (see Methods). To mimic the growth of *Y. pestis* at different temperatures, we adapted the previous approach of using two different biomass objective functions (BOFs) that are designed to mimic the cellular requirements for replication under these conditions [17]. We conducted single-gene knockout analyses under each condition (see Methods), which resulted in the prediction of 149 and 146 metabolic genes as essential at 25 °C or 37 °C, respectively. All of the genes predicted to be essential at 37 °C were also essential at 25 °C, while there were three essential genes that were specific to 25 °C (YPO1139, YPO2063 and YPO3632). Given this overlap, we have conducted additional analyses described below only using the 25 °C BOF.

**Fig. 2:**
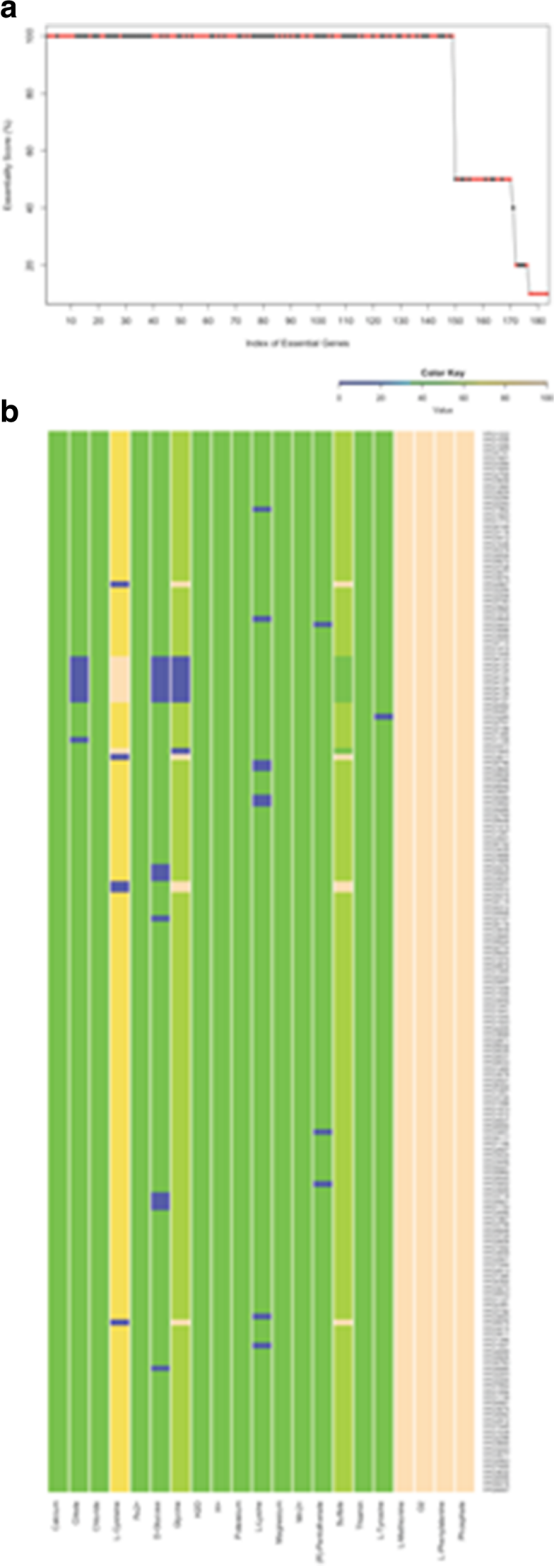
The gene essentiality score and dependence of gene essentiality on specific media components found in BCS media. **a** Gene essentiality score based on media derived from BCS recipe. The x-axis lists all genes predicted as essential at least in one media, while the y-axis shows their essentiality score, calculated as the percentage of the media in which they are predicted to be essential. The overlap with experimentally identified genes in BAB broth media is marked in red. **b** A heat map showing media, in which a given gene was predicted to be essential, in terms of its components. The y-axis lists all genes predicted as essential at least in one media, while the x-axis shows media components. The colored boxes at each x, y location indicate the fraction of media component (x-axis) in all the media where that gene (y-axis) was essential. As shown in the color key, *beige* (*blue*) indicates all media, in which a gene was essential, containing (lacking) a specific media component

When we compared the 149 genes predicted by the model to be essential at 25 °C and the experimentally identified essential gene sets, we found that only 67 genes were common between them. As environment can influence the gene essentiality [56, 57], the discrepancy between the experimentally identified and the model predicted essential genes could be due to the use of different culture media for the transposon mutagenesis experiment (BAB broth medium) and in the metabolic model (BCS medium [17]). Since we do not know the exact chemical composition of the BAB broth medium, we have repeated the gene-essentiality analysis of the metabolic model using a large number of diverse media compositions. As a tractable starting point, we generated all possible combinations among the 17 BCS medium components, resulting in 131,072 media (see Methods). These media produced different essential gene set predictions. The media that produced the highest number of predicted essential genes also resulted in the highest overlap with the experimental data (Fig. 2a), and the number of essential genes decreased with the number of media components present (Fig. 2b). Using this data, we predicted an “essentiality score” for each gene, that is, the percentage of media where a given gene is found to be essential (Additional file 4: Fig. S1A). This revealed that some genes became essential only under media that was lacking specific media elements (Additional file 4: Fig. S1B). For example, a combined lack of glucose, glycine, and citrate revealed that a set of 8 genes became essential (Additional file 4: Fig. S1B). We then went on to perform a broader exploration of the space of possible media by generating over 2 million random media using the 281 exchange reactions in the model (see Methods). We found that several such randomly generated media produced a broader range of essential genes compared to the BCS medium and combinations thereof (Additional file 5: Figure S2). We combined both media sets and re-calculated the “essentiality score” for each gene. We found a set of 78 genes with an essentiality score of 100 (i.e. they were essential in all computationally tested media). Of these, 69.23% (i.e. 54 genes) were overlapping with our experimentally identified essential genes under the BAB broth medium.

We have then identified a list of “core” essential genes by repeating the computational gene knockout analysis under a maximally-rich medium, implemented in the model by enabling uptake fluxes for all the exchange reactions (see Methods). This maximally-rich medium represents an environment with all the media components that *Y. pestis* has access to (according to the model). Therefore, the essential genes identified under this condition will theoretically be essential in all other media compositions. This analysis identified 78 “core” essential genes. These 78 genes are identical to the essential genes with an essentiality score of 100 identified in the above analysis, indicating that our randomly selected media provided a good coverage of the diversity of rich media.

### The core essential genes of *Y. pestis* CO92

Taken together, our experimental results, their comparison with similar previous studies from other *Y. pestis* strains, and our computational analysis of a metabolic model under different environments, indicate a significant context dependency of gene essentiality. This is in line with the theoretical expectations [56, 57] and also fits the intuitive view that certain genes will only be required under certain environments. However, our analyses also highlight that a core set of genes are predicted to be essential in a broad range of environments and are also identified in our experiments and in other experimental studies.

In particular, we can conclude that the set of essential genes predicted both by the presented computational and experimental analyses (54 genes, Additional file 6: Table S4) correspond to genes that are particularly important for the growth of *Y. pestis*. The gene ontology (according to COG groupings) showed that the majority of these genes are related to transport and metabolism of various components, with coenzymes being the most numerous (Table 2). Of the genes we have identified, many are involved in the biosynthesis of fatty acids and lipids. The *fabG* (YPO1599), *fabD* (YPO1598), *accA* (YPO1060), *accB* (YPO3659) *accC* (YPO3658) and *accD* (YPO2768) genes are located in the fatty acid biosynthesis pathway which has previously been identified as a target for antimicrobials [64, 65]. Although the reactions catalyzed by enzymes in this pathway are similar to those found in mammals, there are structural differences in the enzymes involved. This allows the targeting of the bacterial enzymes as for example in Isoniazid, which is used to treat tuberculosis, and triclosan, a broad spectrum antibacterial, both of which target the FabI step of fatty acid synthesis [65]. In lipid A biosynthesis, entire pathways (*lpxA;*YPO1056, *lpxB:*YPO1057, *lpxD;*YPO1054, l*pxH;*YPO3075 and *lpxK;*YPO1396) were identified as essential, although some bacterial species are known to tolerate mutations in lipid A biosynthesis [66, 67].

**Table 2:** Gene ontology of core essential genes

COG category	No of genes	COG function
F	5	nucleotide transport and metabolism
G	3	carbohydrate transport and metabolism
H	17	coenzyme transport and metabolism
I	9	lipid transport and metabolism
M	14	cell wall/membrane/envelope biogenesis
P	1	inorganic ion transport and metabolism
V	1	defence mechanisms
HI	1	coenzyme transport and metabolism, lipid transport and metabolism
GM	1	carbohydrate transport and metabolism, cell wall/membrane/envelope biogenesis
IQ	1	lipid transport and metabolism, secondary metabolites biosynthesis, transport and catabolism
IQR	1	lipid transport and metabolism, secondary metabolites biosynthesis, transport and catabolism, general function prediction only

### Re-assessment of reported gene essentiality data

We have previously reported the experimental validation of selected essential genes [14] and here we interpret this data in the context of our findings here (Table 3). The first gene selected is *accA*, encoding acetyl-coA carboxylase, involved in biosynthesis of malonyl-coA, which is the precursor for fatty acid biosynthesis and already identified as a versatile antimicrobial target in many species [68]. This gene is also in the metabolic model and was identified as “super” essential, as described above. Two genes (*ispG*, *spoT*) were in the model, but identified as possibly non-essential under some environments, and one gene (*trmD*) is not contained in the metabolic model. Of these, the products of genes *spoT* and *trmD* are involved in the regulation of pppGp pools and tRNA production, and mutation of *spoT* has been shown to attenuate *Y. pestis* [62]. The gene product of *ispG* is a diphosphate synthase found in the biosynthesis of isopentenyl diphosphate, which takes a junctional role in pathways leading to the production of longer chain carbohydrates, including terpenes, and mevalonate. For each gene, rhamnose-inducible/glucose-repressible mutants were made, and tested by growth in liquid culture or on solid medium (Table 3). This assay confirmed the essential nature of all the five genes for growth on agar plates, confirming the results of the TraDIS screen. The *accA* mutant was unable to grow on agar or in broth, while the other three mutants were unable to grow on agar, but were able to grow in broth. These results confirm the conditional essential nature of *ispG* and *spoT*, validating both the TraDIS screen and showing the usefulness of integration with the computational approach. Further physiological and infection studies are required to test the potential value of these and other identified genes as novel antibiotic targets.

**Table 3:** Experimental confirmation of essentiality

Gene number	Gene name	Phenotype
YPO1060	*accA*	Essential in broth and on agar
YPO3293	*trmD*	Not essential in broth. Essential on solid media.
YPO2879	*ispG*	Not essential in broth. Essential on solid media.
YPO0038	*spot*	Not essential in broth. Essential on solid media.

In summary we performed and integrated experimental and computational study to identify essential genes in *Y. pestis* CO92. This approach identified genes that are differentially essential under 37 °C and 25 °C, and under our experimental conditions. Our integrated approach highlighted environment-dependency of essential genes and predicted a small subset of genes that are core essential, in that their deletion is expected to impair growth under a broad range of conditions. As expected from this prediction, this core essential subset was mostly identified as essential in all experimental studies conducted on *Y. pestis,* including this study. These gene products are target for the development of novel disease interventions.

This integrated study also highlights the clear environmental dependency of essential genes. In the context of metabolism, this finding makes intuitive sense and shows the relevance of media components and cellular biomass composition for enzymatic requirements of the cell. Under different conditions, both media supplements and cellular composition are expected to change, rendering some of the metabolic pathways and enzymes obsolete or highly required. While our metabolic modelling approach can capture some of this variation in enzyme essentiality, it still does not provide a perfect match with experimental findings. A similar result of partial overlap was reported when comparing the essential genes predicted by metabolic modelling with essential genes identified using a whole genome mutagenesis approach in *Neisseria meningitidis* [46]. These discrepancies reflect that either the genome-scale metabolic models still miss key parts of metabolic pathways, or their assumptions on biomass composition are incomplete. The latter aspect could be explored in future studies by the computational exploration of different biomass composition constructions, while the former would be expected to improve with further physiological studies and genome annotations.

## Conclusion

Using an integrated computational and experimental approach we have identified genes which are required for growth at 37 °C and under a broad range of environments. This work is important because we identify a number of candidate virulence factors in *Y. pestis* which have not previously been reported. These may be important targets for drug discovery or vaccine development. At a broader level our work is important because we demonstrate that the repertoire of essential genes identified using whole genome mutagenesis, now widely reported in a range of pathogens, is a fluid concept which is dependent on small changes in the environment.

## Additional files

Additional file 1: Table S1. Sequences of adapters used during library preparation in this study. (DOCX 11 kb)

Additional file 2: Table S2. Primer sequences used during preparation of libraries for sequencing (DOCX 12 kb)

Additional file 3: Table S3. Essential genes identified in different strains of *Y. pestis* by different methods. Genes in KIM10 identified in in vitro expansion of a *himar1*-derived transposon library grown on TB agar containing 2.5 mM CaCl_2_ and 25 μg/ml zeocin at 37 °C, then analysed using the Hidden Markov model (50). Genes in CO92 identified after in vitro growth in BAB broth at both 28 °C and 37 °C, then analysed using the DEM algorithm (14) (DOCX 48 kb)

Additional file 4: Figure S1. Analysis of gene essentiality using the genome-scale model and 2 million randomly generated media. **A.** Number of essential genes (blue bars; y-axis) predicted by the model using a specific random media composition derived from the available exchange reactions in the model (x-axis). The overlap with experimentally identified genes in BAB broth media is shown as well (red bars). **B.** Number of essential genes (y-axis) predicted by the model using a specific media composition that is containing a given number of components of the available exchange reactions in the model (as shown in the x-axis). (TIFF 156 kb)

Additional file 5: Figure S2. Analysis of gene essentiality using the genome-scale model and 2 million randomly generated media (using available exchange reactions in the model). The x-axis list the number of genes identified as essential in a set of media (y-axis). (TIFF 95 kb)

Additional file 6: Table S4. Overlap between experimentally identified and computationally predicted essential genes. Lines 1–54 details genes that are predicted to be essential in all computationally tested media (100% essentiality score) and are also experimentally identified to be an essential gene under the BAB broth medium. Lines 55–78 details genes that are predicted to be essential in all computationally tested media (100% essentiality score) but not experimentally identified to be an essential gene under the BAB broth medium. The gene information was collected from http://www.genome.jp on October 18 2016. (DOCX 17 kb)

## Abbreviations

BAB: Blood agar base
BOF: Biomass objective function; BCS
Bp: Base pairs
Cmk: Cytidine monophosphate kinase
COGs: Cluster of orthologous groups
DEG: Database of essential genes
dNTPs: Nucleoside triphosphates
FBA: Using flux balance analysis
HITS: High-throughput insertion tracking by deep sequencing
HMM: Hidden markov method
INSeq: Insertion sequencing
PCR: Polymerase chain reaction
TBE: tris-borate-EDTA
tmRNA: Transfer-messenger RNA
Tn-seq: Transposon sequencing
TraDIS: Transposon-directed insertion site sequencing
