# Supplementary material for: An integrated computational-experimental approach reveals Yersinia pestis genes essential across a narrow or a broad range of environmental conditions
Source: BMC Microbiol. 2017 Jul 21;17:163. doi: 10.1186/s12866-017-1073-8 (PMC5521123; doi:10.1186/s12866-017-1073-8)
Supplement: Supplementary file 1 — Sequences of adapters used during library preparation in this study. (DOCX 11 kb) [file 12866_2017_1073_MOESM1_ESM.docx]

**Table S1**. Sequences of adapters used during library preparation in this study.

| Adapter | Sequence | Comment |
| --- | --- | --- |
| **Ind_Ad_T** | ACACTCTTTCCCTACACGACGCTCTTCCGATC*T | *Phosphorothioate |
| **Ind_Ad_B** | pGATCGGAAGAGCGGTTCAGCAGGAATGCCGAGACCGATCTC | Phosphorylated |
