# Supplementary material for: An integrated computational-experimental approach reveals Yersinia pestis genes essential across a narrow or a broad range of environmental conditions
Source: BMC Microbiol. 2017 Jul 21;17:163. doi: 10.1186/s12866-017-1073-8 (PMC5521123; doi:10.1186/s12866-017-1073-8)
Supplement: Supplementary file 2 — Primer sequences used during preparation of libraries for sequencing (DOCX 12 kb) [file 12866_2017_1073_MOESM2_ESM.docx]

**Table S2** Primer sequences used during preparation of libraries for sequencing

| **Primer** | **Sequence** | **Comment** |
| --- | --- | --- |
| **PE_PCR_V3.3** | **CAAGCAGAAGACGGCATACGA**GATCGGTACACTCTTTCCCTACACGACGCTCTTCCGATCT | Flow cell binding region in bold |
| **Yp EZ_Tn PCR** | **AATGATACGGCGACCACCGAGATCTACAC**ACCTACAACAAAGCTCTCATCAACC | Flow cell binding region in bold |
| **Yp EZ_Tn seq** | TGCAAGCTTCAGGGTTGAGA |  |
