# Supplementary material for: An integrated computational-experimental approach reveals Yersinia pestis genes essential across a narrow or a broad range of environmental conditions
Source: BMC Microbiol. 2017 Jul 21;17:163. doi: 10.1186/s12866-017-1073-8 (PMC5521123; doi:10.1186/s12866-017-1073-8)
Supplement: Supplementary file 3 — Essential genes identified in different strains of Y. pestis by different methods. Genes in KIM10 identified in in vitro expansion of a himar1-derived transposon library grown on TB agar containing 2.5 mM CaCl2 and 25 μg/ml zeocin at 37 °C, then analysed using the Hidden Markov model (50). Genes in CO92 identified after in vitro growth in BAB broth at both 28 °C and 37 °C, then analysed using the DEM algorithm (14) (DOCX 48 kb) [file 12866_2017_1073_MOESM3_ESM.docx]

**Table S3: Essential genes identified in different strains of *Y. pestis* by different methods.** Genes in KIM10 identified in *in vitro* expansion of a *himar1*-derived transposon library grown on TB agar containing 2.5 mM CaCl_2_ and 25 μg/ml zeocin at 37^o^C, then analysed using the Hidden Markov model (50). Genes in CO92 identified after *in vitro* growth in BAB broth at both 28^o^C and 37^o^C, then analysed using the DEM algorithm (14)

| **Essential genes in KIM10 by HMM** | | **Homologue in DEG** | **Essential genes in CO92 at 28^o^C** | | **Essential genes in CO92 at 37^o^C** | |
| --- | --- | --- | --- | --- | --- | --- |
| Gene ID | Gene name |  | Gene ID | Gene name | Gene ID | Gene name |
| y0024 | *pgi* | Yes | YPO3718 | *pgi* |  |  |
| y0052 | *tpiA* | Yes | YPO0085 | *tpiA* | YPO0085 | *tpiA* |
| y0059 | *pfkA* | Yes | YPO0078 | *pfkA* | YPO0078 | *pfkA* |
| y0069 | *cpxA* | No | YPO0073 | *cpxA* | YPO0073 | *cpxA* |
| y0072 | *cysE* | Yes | YPO0070 | *cysE* | YPO0070 | *cysE* |
| y0083 | *rfaD* | Yes | YPO0058 | *rfaD* | YPO0058 | *rfaD* |
| y0084 | *rfaF* | Yes | YPO0057 | *rfaF* | YPO0057 | *rfaF* |
| y0085 | *rfaC* | Yes | YPO0056 | *rfaC* | YPO0056 | *rfaC* |
| y0086 | *kdtA* | Yes | YPO0055 | *kdtA* | YPO0055 | *kdtA* |
| y0087 | - | No |  |  |  |  |
| y0088 | *coaD* | Yes | YPO0053 | *coaD* | YPO0053 | *coaD* |
| y0091 | *rpmG* | Yes | YPO0051 | *rpmG* | YPO0051 | *rpmG* |
| y0093 | *dfp* | Yes | YPO0048 | *dfp* | YPO0048 | *dfp* |
| y0094 | *dut* | No | YPO0047 | *dut* | YPO0047 | *dut* |
| y0101 | *gmk* | Yes | YPO0040 | *gmk* | YPO0040 | *gmk* |
| y0102 | *rpoZ* | Yes | YPO0039 | *rpoZ* | YPO0039 | *rpoZ* |
| y0103 | *spoT* | Yes | YPO0038 | *spoT* | YPO0038 | *spoT* |
| y0105 | *recG* | Yes | YPO0036 | *recG* | YPO0036 | *recG* |
| y0132 | *sspA* | Yes | YPO3561 | *sspA* | YPO3561 | *sspA* |
| y0133 | *rpsI* | Yes | YPO3562 | *rpsI* | YPO3562 | *rpsI* |
| y0134 | *rplM* | Yes | YPO3563 | *rplM* | YPO3563 | *rplM* |
| y0139 | *degS* | Yes | YPO3568 | *degS* | YPO3568 | *degS* |
| y0140 | *murA* | Yes | YPO3569 | *murA* | YPO3569 | *murA* |
| y0149 | NR | Yes | YPO3577 | NR | YPO3577 | - |
| y0151 | NR | Yes | YPO3579 | *yrbK* | YPO3579 | *yrbK* |
| y0152 | NR | Yes | YPO3580 | NR | YPO3580 | - |
| y0153 | NR | Yes | YPO3581 | *yhbG* | YPO3581 | *yhbG* |
| y0158 | NR | No | YPO3586 | NR | YPO3586 | - |
| y0184 | *tcbA* | No |  |  |  |  |
| y0198 | NR | No |  |  |  |  |
| y0199 | *mreD* | Yes | YPO3667 | *mreD* | YPO3667 | *mreD* |
| y0201 | *mreC* | Yes | YPO3666 | *mreC* | YPO3666 | *mreC* |
| y0202 | *mreB* | Yes | YPO3665 | *mreB* | YPO3665 | *mreB* |
| y0207 | NR | Yes | YPO3660 | *aroQ* | YPO3660 | *aroQ* |
| y0208 | *accB* | Yes | YPO3659 | *accB* | YPO3659 | *accB* |
| y0209 | *accC* | Yes | YPO3658 | *accC* | YPO3658 | *accC* |
| y0254 | NR | No | YPO3616 | NR |  |  |
| y0296 | *ftsN* | Yes |  |  |  |  |
| y0298 | *priA* | Yes | YPO0110 | *priA* | YPO0110 | *priA* |
| y0299 | *rpmE* | Yes | YPO0111 | *rpmE* | YPO0111 | *rpmE* |
| y0321 | *udhA* | Yes |  |  |  |  |
| y0326 | *murI* | Yes | YPO3909 | *murI* |  |  |
| y0340 | NR | No |  |  |  |  |
| y0351 | NR | Yes |  |  |  |  |
| y0356 | *rep* | Yes | YPO3872 | *rep* | YPO3872 | *rep* |
| y0361 | *rho* | Yes | YPO3867 | *rho* |  |  |
| y0364 | *wecB* | Yes |  |  | YPO3864 | *nfrC* |
| y0365 | *wecC* | Yes | YPO3863 | *rffD* |  |  |
| y0366 | *rffG* | Yes | YPO3862 | *rffG* | YPO3862 | *rffG* |
| y0367 | *rffH* | Yes | YPO3861 | *rffH* | YPO3861 | *rffH* |
| y0368 | *wecD* | No | YPO3860 | *rffC* | YPO3860 | *rffC* |
| y0369 | *wecE* | Yes | YPO3859 | *rffA* | YPO3859 | *rffA* |
| y0371 | *wecF* | Yes | YPO3857 | - | YPO3857 | - |
| y0372 | *wecF* | Yes | YPO3856 | *wecF* | YPO3856 | *wecF* |
| y0373 | *wecG* | Yes | YPO3855 | *wecG* | YPO3855 | *wecG* |
| y0380 | *hemD* | Yes | YPO3850 | *hemD* | YPO3850 | *hemD* |
| y0381 | *hemC* | Yes | YPO3849 | *hemC* | YPO3849 | *hemC* |
| y0382 | *cyaA* | Yes | YPO3848 | *cyaA* | YPO3848 | *cyaA* |
| y0385 | *dapF* | Yes | YPO3845 | *dapF* | YPO3845 | *dapF* |
| y0387 | *xerC* | Yes | YPO3843 | *xerC* |  |  |
| y0416 | *ftsY* | Yes | YPO3814 | *ftsY* | YPO3814 | *ftsY* |
| y0417 | *ftsE* | Yes |  |  |  |  |
| y0418 | *ftsX* | Yes |  |  |  |  |
| y0419 | *rpoH* | Yes | YPO3811 | *rpoH* |  |  |
| y0449 | *ubiE* | Yes | YPO3781 | *ubiE* | YPO3781 | *ubiE* |
| y0451 | *ubiB* | Yes | YPO3779 | *aarF* | YPO3779 | *aarF* |
| y0452 | NR | No | YPO3778 | *tatA* | YPO3778 | *tatA* |
| y0453 | NR | No |  |  |  |  |
| y0454 | NR | Yes | YPO3776 | *tatC* | YPO3776 | *tatC* |
| y0459 | *hemB* | Yes | YPO3771 | *hemB* | YPO3771 | *hemB* |
| y0461 | NR | Yes | YPO3769 | - |  |  |
| y0462 | *fre* | No | YPO3768 | *fadI* | YPO3768 | *fadI* |
| y0468 | *trkH* | Yes | YPO3762 | *trkH* | YPO3762 | *trkH* |
| y0469 | *hemG* | Yes | YPO3761 | *hemG* | YPO3761 | *hemG* |
| y0471 | *murB* | Yes | YPO3760 | *murB* |  |  |
| y0472 | *birA* | Yes |  |  |  |  |
| y0473 | *coaA* | Yes | YPO3758 | *coaA* | YPO3758 | *coaA* |
| y0478 | *secE* | Yes | YPO3753 | *secE* | YPO3753 | *secE* |
| y0479 | *nusG* | Yes | YPO3752 | *nusG* | YPO3752 | *nusG* |
| y0480 | *rplK* | Yes | YPO3751 | *rplK* | YPO3751 | *rplK* |
| y0481 | *rplA* | Yes | YPO3750 | *rplA* | YPO3750 | *rplA* |
| y0482 | *rplJ* | Yes | YPO3749 | *rplJ* | YPO3749 | *rplJ* |
| y0483 | NR | Yes | YPO3748 | *rplL* | YPO3748 | *rplL* |
| y0484 | *rpoB* | Yes | YPO3747 | *groN* | YPO3747 | *groN* |
| y0485 | *rpoC* | Yes | YPO3746 | *rpoC* | YPO3746 | *rpoC* |
| y0487 | *thiH* | No |  |  |  |  |
| y0488 | *thiG* | Yes |  |  |  |  |
| y0489 | *thiF* | Yes |  |  |  |  |
| y0490 | *thiE* | Yes |  |  |  |  |
| y0491 | *thiC* | Yes |  |  |  |  |
| y0496 | *hemE* | Yes | YPO3734 | *hemE* | YPO3734 | *hemE* |
| y0516 | NR | No |  |  |  |  |
| y0536 | NR | Yes |  |  |  |  |
| y0558 | *terC* | No | YPO0297 | *terC* |  |  |
| y0559 | *terD* | Yes |  |  |  |  |
| y0569 | *ubiA* | Yes | YPO0311 | *ubiA* | YPO0311 | *ubiA* |
| y0570 | *plsB* | Yes | YPO0312 | *plsB* |  |  |
| y0572 | *lexA* | Yes | YPO0314 | *lexA* | YPO0314 | *lexA* |
| y0577 | *dnaB* | Yes | YPO0320 | *dnaB* | YPO0320 | *dnaB* |
| y0578 | *alr* | Yes | YPO0321 | *alr* | YPO0321 | *alr* |
| y0582 | *ssb* | Yes | YPO0325 | *ssb* | YPO0325 | *ssb* |
| y0597 | NR | No |  |  |  |  |
| y0608 | *groES* | Yes | YPO0350 | *groES* | YPO0350 | *groES* |
| y0609 | *groEL* | Yes | YPO0351 | *groEL* | YPO0351 | *groEL* |
| y0611 | NR | Yes |  |  |  |  |
| y0612 | *efp* | Yes | YPO0354 | *efp* | YPO0354 | *efp* |
| y0620 | *psd* | Yes | YPO0364 | *psd* | YPO0364 | *psd* |
| y0623 | NR | Yes | YPO0366 | *orn* | YPO0366 | *orn* |
| y0626 | NR | Yes | YPO0369 | NR | YPO0369 | NR |
| y0627 | *amiB* | Yes |  |  |  |  |
| y0630 | *hfq* | Yes |  |  |  |  |
| y0635 | *purA* | Yes |  |  |  |  |
| y0645 | *rpsF* | Yes | YPO3539 | *rpsF* | YPO3539 | *rpsF* |
| y0646 | *rpsR* | Yes | YPO3537 | *rpsR* | YPO3537 | *rpsR* |
| y0663 | *ppa* | Yes | YPO3521 | *ppa* | YPO3521 | *ppa* |
| y0664 | *fbp* | Yes |  |  |  |  |
| y0671 | *ispB* | Yes | YPO3513 | *ispB* | YPO3513 | *ispB* |
| NR | *rplU* | Yes | YPO3512 | *rplU* | YPO3512 | *rplU* |
| y0673 | *rpmA* | Yes | YPO3511 | *rpmA* | YPO3511 | *rpmA* |
| y0675 | *obgE* | Yes | YPO3509 | *obgE* | YPO3509 | *obgE* |
| y0681 | *rrmJ* | Yes | YPO3503 | *rrmJ* | YPO3503 | *rrmJ* |
| y0682 | *hflB* | Yes | YPO3502 | *ftsH* | YPO3502 | *ftsH* |
| y0683 | *folP* | Yes | YPO3501 | *folP* | YPO3501 | *folP* |
| y0684 | *glmM* | Yes | YPO3500 | *glmM* | YPO3500 | *mrsA* |
| y0686 | NR | No | YPO3498 | NR | YPO3498 | NR |
| y0687 | *nusA* | Yes | YPO3497 | *nusA* | YPO3497 | *nusA* |
| y0688 | *infB* | Yes | YPO3496 | *infB* | YPO3496 | *infB* |
| y0689 | *rbfA* | Yes | YPO3495 | *rbfA* | YPO3496 | *rbfA* |
| y0691 | *rpsO* | Yes | YPO3493 | *rpsO* | YPO3493 | *rpsO* |
| y0696 | *deaD* | Yes | YPO3488 | *deaD* |  |  |
| y0712 | - | No |  |  |  |  |
| y0743 | *valS* | Yes | YPO3443 | *valS* | YPO3443 | *valS* |
| y0745 | *holC* | Yes | YPO3442 | *holC* | YPO3442 | *holC* |
| y0747 | NR | No | YPO3440 | NR | YPO3440 | NR |
| y0748 | NR | No | YPO3439 | NR | YPO3439 | NR |
| y0757 | *coaE* | Yes | YPO3430 | *coaE* | YPO3430 | *coaE* |
| y0767 | *aceE* | Yes | YPO3419 | *aceE* | YPO3419 | *aceE* |
| y0768 | *aceF* | Yes | YPO3418 | *aceF* | YPO3418 | *aceF* |
| y0769 | *lpdA* | Yes | YPO3417 | *lpdA* | YPO3417 | *lpdA* |
| y0771 | *acnB* | No | YPO3415 | *acnB* | YPO3415 | *acnB* |
| y0779 | NR | Yes | YPO3407 | *yadF* | YPO3407 | *yadF* |
| y0787 | *folK* | Yes | YPO3400 | *folK* |  |  |
| y0788 | *pcnB* | No |  |  |  |  |
| y0799 | *hemL* | Yes | YPO3389 | *hemL* | YPO3389 | *hemL* |
| y0801 | NR | Yes | YPO3387 | NR | YPO3387 | NR |
| y0805 | *pfs* | Yes | YPO3384 | *mtn* |  |  |
| y0813 | *pyrG* | Yes | YPO3377 | *pyrG* | YPO3377 | *pyrG* |
| y0814 | *eno* | Yes | YPO3376 | *eno* | YPO3376 | *eno* |
| y0827 | *ftsB* | Yes | YPO3362 | *ftsB* | YPO3362 | *ftsB* |
| y0828 | *ispD* | Yes | YPO3361 | *ispD* | YPO3361 | *ispD* |
| y0829 | *ispF* | Yes | YPO3360 | *ispF* |  |  |
| y0833 | *nlpD* | No | YPO3356 | *nlpD* |  |  |
| y0881 | *recA* | Yes | YPO3307 | *recA* | YPO3307 | *recA* |
| y0883 | *alaS* | Yes | YPO3305 | *alaS* | YPO3305 | *alaS* |
| y0884 | *csrA* | Yes | YPO3304 | *csrA* | YPO3304 | *csrA* |
| y0892 | *ffh* | Yes | YPO3296 | *ffh* | YPO3296 | *ffh* |
| y0894 | *rpsP* | Yes | YPO3295 | *rpsP* | YPO3295 | *rpsP* |
| y0895 | *rimM* | Yes | YPO3294 | *rimM* | YPO3294 | *rimM* |
| y0896 | *trmD* | Yes | YPO3293 | *trmD* | YPO3293 | *trmD* |
| y0897 | *rplS* | Yes | YPO3292 | *rplS* | YPO3292 | *rplS* |
| y0911 | NR | Yes | YPO3278 | *yfiO* | YPO3278 | *yfiO* |
| y0916 | *pssA* | Yes | YPO3273 | *pssA* | YPO3273 | *pssA* |
| y0940 | NR | No |  |  |  |  |
| y0947 | *gmhA* | Yes | YPO3243 | *gmhA* |  |  |
| y0993 | *secD* | Yes | YPO3189 | *secD* | YPO3189 | *secD* |
| y0994 | *secF* | Yes | YPO3188 | *secF* | YPO3188 | *secF* |
| y0996 | NR | No |  |  |  |  |
| y0997 | NR | No |  |  |  |  |
| y1000 | *ribD* | Yes | YPO3183 | *ribD* | YPO3183 | *ribD* |
| y1001 | *ribH* | Yes | YPO3182 | *ribE* | YPO3182 | *ribE* |
| y1002 | *nusB* | Yes | YPO3181 | *nusB* | YPO3181 | *nusB* |
| y1003 | *thiL* | Yes | YPO3180 | *thiL* | YPO3180 | *thiL* |
| y1008 | *dxs* | Yes | YPO3177 | *dxs* | YPO3177 | *dxs* |
| y1009 | *ispA* | Yes | YPO3176 | *ispA* | YPO3176 | *ispA* |
| y1018 | *cyoE* | No |  |  |  |  |
| y1020 | *cyoB* | Yes |  |  |  |  |
| y1021 | *cyoA* | Yes |  |  |  |  |
| y1029 | *lon* | Yes | YPO3155 | *Lon* | YPO3155 | *Lon* |
| y1046 | *hha* | No |  |  |  |  |
| y1049 | *acrB* | Yes |  |  |  |  |
| y1060 | *dnaX* | Yes | YPO3122 | *dnaX* | YPO3122 | *dnaX* |
| y1065 | *adk* | Yes | YPO3118 | *adk* | YPO3118 | *adk* |
| y1066 | *hemH* | Yes | YPO3117 | *hemH* | YPO3117 | *hemH* |
| y1067 | *ascD* | Yes |  |  |  |  |
| y1072 | NR | No |  |  |  |  |
| y1074 | NR | No |  |  |  |  |
| y1075 | NR | No |  |  |  |  |
| y1076 | NR | No |  |  |  |  |
| y1082 | NR | Yes |  |  |  |  |
| y1083 | *cpsG* | Yes |  |  |  |  |
| y1084 | NR | No |  |  |  |  |
| y1105 | NR | Yes | YPO3075 | NR | YPO3075 | NR |
| y1106 | *ppiB* | Yes | YPO3074 | *ppiB* | YPO3074 | *ppiB* |
| y1107 | *cysS* | Yes | YPO3073 | *cysS* | YPO3073 | *cysS* |
| y1110 | *folD* | Yes | YPO2824 | *folD* |  |  |
| y1134 | *yapC* | No |  |  |  |  |
| y1136 | NR | No |  |  |  |  |
| y1139 | NR | No |  |  |  |  |
| y1171 | *lipA* | Yes | YPO2598 | *lipA* | YPO2598 | *lipA* |
| y1177 | *mrdB* | Yes | YPO2603 | *rodA* |  |  |
| y1178 | *mrdA* | Yes | YPO2604 | *pbpA* | YPO2604 | *pbpA* |
| y1181 | *nadD* | Yes | YPO2607 | *nadD* | YPO2607 | *nadD* |
| y1182 | *holA* | Yes | YPO2608 | *holA* | YPO2608 | *holA* |
| y1183 | *rlpB* | Yes | YPO2609 | *rplB* | YPO2609 | *rplB* |
| y1184 | *leuS* | Yes | YPO2610 | *leuS* | YPO2610 | *leuS* |
| y1190 | *lnt* | Yes | YPO2616 | *lnt* |  |  |
| y1193 | NR | Yes | YPO2618 | NR |  |  |
| y1196 | *ubiF* | Yes | YPO2621 | *ubiF* | YPO2621 | *ubiF* |
| y1200 | *nagC* | No |  |  |  |  |
| y1205 | *glnS* | Yes | YPO2630 | *glnS* | YPO2630 | *glnS* |
| y1208 | *fur* | Yes | YPO2634 | *fur* | YPO2634 | *fur* |
| y1209 | *fldA* | Yes | YPO2635 | *fldA* | YPO2635 | *fldA* |
| y1250 | NR | No |  |  |  |  |
| y1270 | *phrB* | Yes |  |  |  |  |
| y1290 | *rpoE* | Yes | YPO2711 | *rpoE* | YPO2711 | *rpoE* |
| y1291 | *rseA* | Yes | YPO2712 | *rseA* | YPO2712 | *rseA* |
| y1294 | NR | No |  |  |  |  |
| y1296 | *lepB* | Yes | YPO2717 | *lepB* | YPO2717 | *lepB* |
| y1297 | *rnc* | Yes |  |  |  |  |
| y1298 | *era* | Yes | YPO2719 | *era* |  |  |
| y1300 | *pdxJ* | Yes | YPO2930 | *pdxJ* | YPO2930 | *pdxJ* |
| y1301 | *acpS* | Yes | YPO2929 | *acpS* | YPO2929 | *acpS* |
| y1307 | NR | Yes | YPO2923 | NR | YPO2923 | - |
| y1316 | *nadE* | Yes | YPO2912 | *nadE* | YPO2912 | *nadE* |
| y1322 | *glyA* | Yes |  |  | YPO2907 | *glyA* |
| y1331 | *suhB* | Yes | YPO2899 | *suhB* | YPO2899 | *suhB* |
| y1334 | NR | Yes | YPO2896 | *iscS* |  |  |
| y1348 | NR | No |  |  |  |  |
| y1349 | *ndk* | Yes |  |  | YPO2883 | *ndk* |
| y1352 | NR | Yes |  |  |  |  |
| y1353 | *ispG* | Yes | YPO2879 | *ispG* |  |  |
| y1354 | *hisS* | Yes | YPO2878 | *hisS* | YPO2878 | *hisS* |
| y1355 | NR | No | YPO2877 | *yfgM* | YPO2877 | *yfgM* |
| y1356 | NR | Yes | YPO2876 | *yfgL* | YPO2876 | *yfgL* |
| y1357 | *engA* | Yes | YPO2875 | *engA* | YPO2875 | *engA* |
| y1372 | NR | No |  |  |  |  |
| y1373 | *thiD* | Yes |  |  |  |  |
| y1398 | *ppk* | Yes |  |  |  |  |
| y1410 | NR | No | YPO3071 | NR | YPO3071 | NR |
| y1418 | *dapA* | Yes | YPO3062 | *dapA* | YPO3062 | *dapA* |
| y1426 | NR | No | YPO3054 | - |  |  |
| y1427 | *dapE* | Yes | YPO3053 | *dapE* | YPO3053 | *dapE* |
| y1436 | NR | No |  |  |  |  |
| y1438 | NR | No |  |  |  |  |
| y1451 | *hemF* | Yes |  |  |  |  |
| y1460 | NR | No |  |  |  |  |
| y1467 | *cysU* | Yes |  |  |  |  |
| y1468 | *cysW* | Yes |  |  |  |  |
| y1470 | *cysM* | Yes |  |  |  |  |
| y1485 | *crr* | Yes | YPO2995 | *crr* | YPO2995 | *crr* |
| y1487 | *ptsI* | Yes | YPO2994 | *ptsI* | YPO2994 | *ptsI* |
| y1488 | *ptsH* | Yes | YPO2993 | *ptsH* | YPO2993 | *ptsH* |
| y1491 | *zipA* | Yes | YPO2990 | *zipA* | YPO2990 | *zipA* |
| y1492 | *ligA* | Yes | YPO2989 | *ligA* | YPO2989 | *ligA* |
| y1498 | *gltX* | Yes | YPO2984 | *gltX* | YPO2984 | *gltX* |
| y1522 | NR | No |  |  |  |  |
| y1534 | NR | No |  |  |  |  |
| y1541 | *ecpD* | Yes |  |  |  |  |
| y1582 | NR | Yes |  |  |  |  |
| y1591 | *fabB* | Yes | YPO2757 | *fabB* | YPO2757 | *fabB* |
| y1597 | *pdxB* | Yes |  |  |  |  |
| y1601 | *accD* | Yes | YPO2768 | *accD* | YPO2768 | *accD* |
| y1602 | *folC* | Yes | YPO2769 | *folC* | YPO2769 | *folC* |
| y1603 | *dedD* | No |  |  |  |  |
| y1606 | *ubiX* | Yes | YPO2773 | *ubiX* | YPO2773 | *ubiX* |
| y1630 | *nuoA* | Yes | YPO2555 | *nuoA* | YPO2555 | *nuoA* |
| y1631 | *nuoB* | Yes | YPO2554 | *nuoB* |  |  |
| y1632 | *nuoC* | Yes | YPO2553 | *nuoD* | YPO2553 | *nuoD* |
| y1633 | *nuoE* | Yes | YPO2552 | *nuoE* | YPO2552 | *nuoE* |
| y1634 | *nuoF* | Yes | YPO2551 | *nuoF* | YPO2551 | *nuoF* |
| y1635 | *nuoG* | Yes | YPO2550 | *nuoG* | YPO2550 | *nuoG* |
| y1636 | *nuoH* | Yes | YPO2549 | *nuoH* | YPO2549 | *nuoH* |
| y1637 | *nuoI* | Yes | YPO2548 | *nuoI* | YPO2548 | *nuoI* |
| y1638 | *nuoJ* | Yes | YPO2547 | *nuoJ* | YPO2547 | *nuoJ* |
| y1639 | *nuoK* | Yes | YPO2546 | *nuoK* | YPO2546 | *nuoK* |
| y1640 | *nuoL* | Yes | YPO2545 | *nuoL* | YPO2545 | *nuoL* |
| y1641 | *nuoM* | Yes | YPO2544 | *nuoM* | YPO2544 | *nuoM* |
| y1642 | *nuoN* | Yes | YPO2543 | *nuoN* | YPO2543 | *nuoN* |
| y1652 | NR | No |  |  |  |  |
| y1698 | NR | No | YPO2489 | NR |  |  |
| y1699 | NR | No |  |  |  |  |
| y1702 | NR | No | YPO2485 | NR | YPO2485 | NR |
| y1724 | NR | No | YPO2465 | NR | YPO2465 | NR |
| y1725 | NR | No | YPO2464 | NR | YPO2464 | NR |
| y1726 | NR | No | YPO2463 | NR | YPO2463 | NR |
| y1727 | NR | No | YPO2462 | NR | YPO2462 | NR |
| y1732 | *ypeR* | No |  |  |  |  |
| y1749 | *rne* | Yes |  |  |  |  |
| y1756 | *fabH* | Yes | YPO1597 | *fabH* | YPO1597 | *fabH* |
| y1757 | *fabD* | Yes | YPO1598 | *fabD* | YPO1598 | *fabD* |
| y1758 | *fabG* | Yes | YPO1599 | *fabG* | YPO1599 | *fabG* |
| y1759 | *acpP* | Yes | YPO1600 | *acpP* | YPO1600 | *acpP* |
| y1764 | *tmk* | Yes | YPO1605 | *tmk* | YPO1605 | *tmk* |
| y1765 | *holB* | Yes | YPO1606 | *holB* |  |  |
| y1785 | NR | Yes | YPO1626 | *lolC* | YPO1626 | *lolC* |
| y1786 | *lolD* | Yes | YPO1627 | *lolD* | YPO1627 | *lolD* |
| y1787 | NR | Yes | YPO1628 | *lolE* | YPO1628 | *lolE* |
| y1797 | *purB* | Yes |  |  |  |  |
| y1799 | *mnmA* | Yes | YPO1638 | *mnmA* |  |  |
| y1802 | *icdA* | Yes | YPO1641 | *icdA* |  |  |
| y1845 | NR | Yes |  |  |  |  |
| y1867 | *prc* | Yes | YPO1705 | *prc* | YPO1705 | *prc* |
| y1903 | *thrS* | Yes | YPO2433 | *thrS* | YPO2433 | *thrS* |
| y1904 | *infC* | Yes | YPO2432 | *infC* | YPO2432 | *infC* |
| y1905 | *rplT* | Yes | YPO2430 | *pdzA* | TPO2430 | *pdzA* |
| y1908 | *pheS* | Yes | YPO2429 | *pheS* | YPO2429 | *pheS* |
| y1909 | *pheT* | Yes | YPO2428 | *pheT* | YPO2428 | *pheT* |
| y1910 | *ihfA* | Yes | YPO2427 | *ihfA* | YPO2427 | *ihfA* |
| y1917 | NR | Yes |  |  |  |  |
| y1937 | NR | Yes |  |  |  |  |
| y1940 | NR | No |  |  |  |  |
| y1946 | *ribE* | Yes | YPO2391 | *ribC* |  |  |
| y1965 | *pdxH* | Yes |  |  |  |  |
| y1966 | *tyrS* | Yes | YPO2369 | *tyrS* | YPO2369 | *tyrS* |
| y1974 | *sapD* | Yes | YPO2358 | *sapD* | YPO2358 | *sapD* |
| y1975 | *sapC* | No | YPO2357 | *sapC* | YPO2357 | *sapC* |
| y1976 | *sapB* | No |  |  |  |  |
| y1981 | *pspB* | No |  |  | YPO2350 | *pspB* |
| y1982 | *pspC* | Yes |  |  |  |  |
| y1995 | NR | No |  |  |  |  |
| y2031 | NR | No |  |  |  |  |
| y2040 | NR | Yes | YPO2196 | *ispA* | YPO2196 | *ispA* |
| y2060 | *topA* | Yes | YPO2218 | *topA* | YPO2218 | *topA* |
| y2061 | *cysB* | Yes |  |  |  |  |
| y2062 | NR | No |  |  |  |  |
| y2064 | *ribA* | Yes | YPO2222 | *ribA* | YPO2222 | *ribA* |
| y2067 | NR | Yes | YPO2225 | NR |  |  |
| y2081 | NR | Yes | YPO2240 | NR | YPO2240 | NR |
| y2082 | NR | Yes | YPO2241 | NR | YPO2241 | NR |
| y2083 | *rnfD* | Yes | YPO2242 | NR | YPO2242 | NR |
| y2086 | NR | Yes | YPO2245 | NR | YPO2245 | NR |
| y2087 | NR | No | YPO2246 | NR | YPO2246 | NR |
| y2146 | *hns* | Yes | YPO2175 | *hns* | YPO2175 | *hns* |
| y2152 | NR | No |  |  |  |  |
| y2156 | *topB* | Yes |  |  |  |  |
| y2165 | *gapA* | Yes | YPO2157 | *gapA* | YPO2157 | *gapA* |
| y2177 | *fadR* | No |  |  |  |  |
| y2178 | NR | Pseudo |  |  |  |  |
| y2190 | NR | No |  |  |  |  |
| y2192 | NR | No |  |  |  |  |
| y2234 | *minE* | Yes | YPO2076 | *minE* | YPO2076 | *minE* |
| y2252 | *ruvB* | Yes | YPO2058 | *ruvB* | YPO2058 | *ruvB* |
| y2258 | *aspS* | Yes | YPO2053 | *aspS* | YPO2053 | *aspS* |
| y2266 | *argS* | Yes | YPO2046 | *argS* | YPO2046 | *argS* |
| y2267 | - | No |  |  |  |  |
| y2269 | *mviN* | Yes | YPO2043 | *mviN* |  |  |
| y2286 | *kdsA* | Yes | YPO2021 | *kdsA* | YPO2021 | *kdsA* |
| y2289 | *hemK* | Yes |  |  |  |  |
| y2290 | *prfA* | Yes | YPO2017 | *prfA* | YPO2017 | *prfA* |
| y2291 | *hemA* | Yes | YPO2016 | *hemA* | YPO2016 | *hemA* |
| y2292 | *lolB* | Yes | YPO2015 | *lolB* | YPO2015 | *lolB* |
| y2293 | *ipk* | Yes | YPO2014 | *ipk* | YPO2014 | *ipk* |
| y2295 | *prsA* | Yes | YPO2013 | *prs* | YPO2013 | *prs* |
| y2297 | *pth* | Yes | YPO2011 | *pth* | YPO2011 | *pth* |
| y2298 | NR | Yes |  |  |  |  |
| y2315 | NR | No |  |  |  |  |
| y2324 | NR | No |  |  |  |  |
| y2396 | *ybtQ* | Yes |  |  |  |  |
| y2397 | *ybtP* | Yes |  |  |  |  |
| y2399 | *irp2* | Yes |  |  |  |  |
| y2422 | NR | No |  |  |  |  |
| y2423 | NR | No |  |  |  |  |
| y2441 | *pgsA* | Yes | YPO1867 | *pgsA* | YPO1867 | *pgsA* |
| y2524 | *ftn* | No |  |  |  |  |
| y2554 | NR | No |  |  |  |  |
| y2626 | *hisA* | Yes |  |  |  |  |
| y2644 | *asmA* | No |  |  |  |  |
| y2647 | *mrp* | Yes |  |  |  |  |
| y2648 | *metG* | Yes | YPO1522 | *metG* | YPO1522 | *metG* |
| y2664 | *folE* | Yes | YPO1505 | *folE* | YPO1505 | *folE* |
| y2669 | NR | No |  |  |  |  |
| y2672 | NR | No |  |  |  |  |
| y2677 | NR | Yes |  |  |  |  |
| y2678 | NR | No |  |  |  |  |
| y2715 | *fabZ* | Yes |  |  |  |  |
| y2723 | NR | Yes | YPO1447 | NR | YPO1447 | NR |
| y2739 | NR | Yes |  |  |  |  |
| y2740 | *fabA* | Yes | YPO1430 | *fabA* | YPO1430 | *fabA* |
| y2758 | *asnC* | Yes | YPO1412 | *asnS* | YPO1412 | *asnS* |
| y2765 | *mukB* | Yes | YPO1405 | *mukB* | YPO1405 | *mukB* |
| y2766 | *mukE* | Yes | YPO1404 | *mukE* |  |  |
| y2767 | *mukF* | Yes | YPO1403 | *mukF* | YPO1403 | *mukF* |
| y2772 | *kdsB* | Yes | YPO1400 | *kdsB* | YPO1400 | *kdsB* |
| y2776 | *lpxK* | Yes | YPO1396 | *lpxK* | YPO1396 | *lpxK* |
| y2777 | *msbA* | Yes | YPO1395 | *msbA* | YPO1395 | *msbA* |
| y2781 | *rpsA* | Yes | YPO1392 | *rpsA* | YPO1392 | *rpsA* |
| y2782 | *cmk* | Yes |  |  | YPO1391 | *cmk* |
| y2784 | *serC* | Yes |  |  |  |  |
| y2796 | *serS* | Yes | YPO1379 | *serS* | YPO1379 | *serS* |
| y2798 | *lolA* | Yes | YPO1377 | *lolA* | YPO1377 | *lolA* |
| y2800 | *ftsK* | Yes |  |  |  |  |
| y2802 | *trxB* | Yes |  |  |  |  |
| y2807 | *infA* | Yes | YPO1370 | *infA* | YPO1370 | *infA* |
| y2881 | *psaB* | Yes |  |  |  |  |
| y2886 | *fruK* | Yes |  |  |  |  |
| y2920 | *rplT* | Yes | YPO1264 | *rplY* | YPO1264 | *rplY* |
| y2954 | NR | No | YPO1233 | NR | YPO1233 | NR |
| y2966 | *ompC* | Yes |  |  |  |  |
| y2970 | *rcsB* | Yes |  |  |  |  |
| y2972 | *gyrA* | Yes | YPO1216 | *gyrA* |  |  |
| y2973 | *ubiG* | Yes | YPO1215 | *ubiG* | YPO1215 | *ubiG* |
| y3016 | NR | No |  |  |  |  |
| y3048 | *gpmA* | Yes |  |  |  |  |
| y3054 | *pal* | Yes | YPO1125 | *pal* | YPO1125 | *pal* |
| y3055 | *tolB* | Yes | YPO1124 | *tolB* | YPO1124 | *tolB* |
| y3056 | *tolA* | Yes | YPO1123 | *tolA* | YPO1123 | *tolA* |
| y3057 | *tolR* | Yes | YPO1121 | *tolR* | YPO1121 | *tolR* |
| y3058 | *tolQ* | Yes | YPO1122 | *tolQ* | YPO1122 | *tolQ* |
| y3064 | *sucD* | Yes | YPO1116 | *sucD* | YPO1116 | *sucD* |
| y3065 | *sucC* | Yes | YPO1115 | *sucC* | YPO1115 | *sucC* |
| y3066 | *sucB* | Yes | YPO1114 | *sucB* | YPO1114 | *sucB* |
| y3067 | *sucA* | Yes | YPO1113 | *sucA* | YPO1113 | *sucA* |
| y3068 | *sdhB* | Yes | YPO1112 | *sdhB* | YPO1112 | *sdhB* |
| y3069 | *sdhA* | Yes | YPO1111 | *sdhA* | YPO1111 | *sdhA* |
| y3070 | *sdhD* | Yes | YPO1110 | *sdhD* | YPO1110 | *sdhD* |
| y3071 | *sdhC* | Yes | YPO1109 | *sdhC* | YPO1109 | *sdhC* |
| y3072 | *gltA* | Yes | YPO1108 | *gltA* | YPO1108 | *gltA* |
| y3073 | *grpE* | Yes | YPO1107 | NR | YPO1107 | NR |
| y3074 | *ppnK* | Yes | YPO1106 | NR | YPO1106 | NR |
| y3076 | *smpA* | Yes |  |  |  |  |
| y3077 | NR | Yes | YPO1103 | NR | YPO1103 | NR |
| y3078 | NR | Yes |  |  | YPO1102 | NR |
| y3079 | *smpB* | Yes |  |  | YPO1101 | *smpB* |
| y3082 | NR | No |  |  |  |  |
| y3083 | NR | No |  |  |  |  |
| y3094 | *dnaQ* | Yes | YPO1082 | *dnaQ* | YPO1082 | *dnaQ* |
| y3109 | *proS* | Yes | YPO1068 | *proS* | YPO1068 | *proS* |
| y3117 | *tilS* | Yes | YPO1062 | NR | YPO1062 | NR |
| y3119 | *accA* | Yes | YPO1060 | *accA* | YPO1060 | *accA* |
| y3120 | *dnaE* | Yes | YPO1059 | *dnaE* | YPO1059 | *dnaE* |
| y3122 | *lpxB* | Yes | YPO1057 | *lpxB* | YPO1057 | *lpxB* |
| y3123 | *lpxA* | Yes | YPO1056 | *lpxA* | YPO1056 | *lpxA* |
| y3124 | *fabZ* | Yes | YPO1055 | *fabZ* | YPO1055 | *fabZ* |
| y3125 | *lpxD* | Yes | YPO1054 | *lpxD* | YPO1054 | *lpxD* |
| y3126 | *ompH* | Yes | YPO1053 | *ompH* | YPO1053 | *ompH* |
| y3127 | NR | Yes | YPO1052 | NR | YPO1052 | NR |
| y3128 | NR | Yes | YPO1051 | NR | YPO1051 | NR |
| y3129 | *cdsA* | Yes | YPO1050 | *cdsA* | YPO1050 | *cdsA* |
| y3130 | NR | Yes | YPO1049 | *rth* | YPO1049 | *rth* |
| y3131 | NR | Yes | YPO1048 | *dxr* | YPO1048 | *dxr* |
| y3133 | *frr* | Yes | YPO1047 | *rrf* | YPO1047 | *rrf* |
| y3134 | *pyrH* | Yes | YPO1046 | *pyrH* | YPO1046 | *pyrH* |
| y3135 | *tsf* | Yes | YPO1045 | *tsf* | YPO1045 | *tsf* |
| y3137 | *rpsB* | Yes | YPO1044 | *rpsB* | YPO1044 | *rpsB* |
| y3139 | *glnD* | Yes |  |  |  |  |
| y3140 | *dapD* | Yes | YPO1041 | *dapD* |  |  |
| y3164 | *recB* | Yes |  |  | YPO1020 | *recB* |
| y3166 | *recC* | Yes |  |  |  |  |
| y3171 | *thyA* | Yes | YPO0783 | *thyA* | YPO0783 | *thyA* |
| y3172 | *lgt* | Yes | YPO0784 | *lgt* | YPO0784 | *lgt* |
| y3174 | NR | Yes | YPO0786 | NR | YPO0786 | NR |
| y3194 | NR | No |  |  |  |  |
| y3195 | NR | Yes |  |  |  |  |
| y3197 | NR | No |  |  |  |  |
| y3198 | NR | No |  |  |  |  |
| y3199 | NR | No |  |  |  |  |
| y3200 | NR | No |  |  |  |  |
| y3272 | *lysS* | Yes | YPO0888 | *lysS* | YPO0888 | *lysS* |
| y3273 | *prfB* | Yes | YPO0889 | *prfB* | YPO0889 | *prfB* |
| y3276 | *xerD* | Yes |  |  | YPO0892 | *xerD* |
| y3283 | NR | Yes | YPO0898 | NR |  |  |
| y3295 | *visC* | No | YPO0908 | *visC* | YPO0908 | *visC* |
| y3296 | *ubiH* | Yes | YPO0909 | *ubiH* | YPO0909 | *ubiH* |
| y3302 | *rpiA* | Yes | YPO0915 | *rpiA* |  |  |
| y3307 | *fba* | Yes | YPO0920 | *fbaA* | YPO0920 | *fbaA* |
| y3308 | *pgk* | Yes | YPO0921 | *pgk* | YPO0921 | *pgk* |
| y3310 | *tktA* | Yes | YPO0926 | *tktA* | YPO0926 | *tktA* |
| y3313 | *speA* | Yes |  |  |  |  |
| y3314 | *metK* | Yes | YPO0931 | *metK* | YPO0931 | *metK* |
| y3321 | *gshB* | Yes | YPO0935 | *gshB* | YPO0935 | *gshB* |
| y3333 | NR | No |  |  |  |  |
| y3341 | *mltC* | No |  |  |  |  |
| y3352 | NR | No |  |  |  |  |
| y3353 | NR | No |  |  |  |  |
| y3394 | *senA* | No |  |  |  |  |
| y3404 | NR | No |  |  |  |  |
| y3467 | NR | No |  |  |  |  |
| y3488 | NR | No |  |  |  |  |
| y3490 | NR | No |  |  |  |  |
| y3493 | NR | No |  |  |  |  |
| y3504 | *sufI* | Yes | YPO0673 | *sufI* |  |  |
| y3505 | NR | No |  |  |  |  |
| y3506 | *plsC* | No | YPO0672 | *parF* | YPO0672 | *parF* |
| y3507 | *parC* | Yes | YPO0671 | *parC* |  |  |
| y3511 | NR | Yes | YPO0668 | *parE* | YPO0668 | *parE* |
| y3516 | *tolC* | Yes |  |  |  |  |
| y3520 | *ribB* | Yes | YPO0658 | *ribB* | YPO0658 | *ribB* |
| y3521 | NR | No |  |  |  |  |
| y3524 | NR | Yes | YPO0654 | *rfaE* | YPO0654 | *rfaE* |
| y3529 | *cca* | Yes | YPO0650 | *cca* |  |  |
| y3531 | *folB* | Yes | YPO0648 | *folB* | YPO0648 | *folB* |
| y3533 | NR | No |  |  |  |  |
| y3534 | NR | Yes | YPO0646 | *gcp* | YPO0646 | *gcp* |
| y3536 | *dnaG* | Yes | YPO0644 | *dnaG* | YPO0644 | *dnaG* |
| y3537 | *rpoD* | Yes | YPO0643 | *rpoD* | YPO0643 | *rpoD* |
| y3538 | NR | No |  |  |  |  |
| y3579 | NR | No |  |  |  |  |
| y3617 | *secA* | Yes | YPO0564 | *secA* |  |  |
| y3619 | NR | No | YPO0562 | NR | YPO0562 | NR |
| y3620 | *lpxC* | Yes | YPO0561 | *lpxC* |  |  |
| y3621 | *ftsZ* | Yes | YPO0560 | *ftsZ* | YPO0560 | *ftsZ* |
| y3622 | *ftsA* | Yes | YPO0559 | *ftsA* | YPO0559 | *ftsA* |
| y3623 | *ftsQ* | Yes | YPO0558 | *ftsQ* | YPO0558 | *ftsQ* |
| y3624 | *ddl* | Yes | YPO0557 | *ddlB* | YPO0557 | *ddlB* |
| y3625 | *murC* | Yes | YPO0556 | *murC* | YPO0556 | *murC* |
| y3626 | *murG* | Yes | YPO0555 | *murG* | YPO0555 | *murG* |
| y3627 | *ftsW* | Yes | YPO0554 | *ftsW* | YPO0554 | *ftsW* |
| y3628 | *murD* | Yes | YPO0553 | *murD* | YPO0553 | *murD* |
| y3629 | *mraY* | Yes | YPO0552 | *murX* | YPO0552 | *murX* |
| y3630 | *murF* | Yes | YPO0551 | *murF* | YPO0551 | *murF* |
| y3631 | *murE* | Yes | YPO0550 | *murE* | YPO0550 | *murE* |
| y3632 | *ftsI* | Yes | YPO0549 | *ftsI* | YPO0549 | *ftsI* |
| y3635 | NR | Yes |  |  |  |  |
| y3680 | *imp* | Yes | YPO0495 | *imp* | YPO0495 | *imp* |
| y3681 | *surA* | Yes | YPO0494 | *surA* | YPO0494 | *surA* |
| y3682 | *pdxA* | Yes |  |  |  |  |
| y3688 | *folA* | Yes | YPO0486 | *folA* | YPO0486 | *folA* |
| y3694 | *dapB* | Yes | YPO0480 | *dapB* | YPO0480 | *dapB* |
| y3697 | *ispH* | Yes | YPO0477 | *ispH* | YPO0477 | *ispH* |
| y3699 | *lspA* | Yes | YPO0476 | *lspA* | YPO0476 | *lspA* |
| y3700 | *ileS* | Yes | YPO0475 | *ileS* | YPO0475 | *ileS* |
| y3701 | *ribF* | Yes | YPO0474 | *ribF* | YPO0474 | *ribF* |
| y3702 | *rpsT* | Yes | YPO0472 | *rpsT* | YPO0472 | *rpsT* |
| y3705 | *dnaJ* | Yes | YPO0469 | *dnaJ* | YPO0469 | *dnaJ* |
| y3706 | *dnaK* | Yes | YPO0468 | *dnaK* |  |  |
| y3708 | - | No |  |  |  |  |
| y3738 | *serB* | Yes |  |  |  |  |
| y3750 | NR | No |  |  |  |  |
| y3752 | *holD* | Yes |  |  |  |  |
| y3790 | NR | No |  |  |  |  |
| y3791 | NR | No |  |  |  |  |
| y3792 | NR | No |  |  |  |  |
| y3804 | *glnA* | Yes | YPO0024 | *glnA* | YPO0024 | *glnA* |
| y3809 | *engB* | Yes | YPO0019 | *engB* | YPO0019 | *engB* |
| y3811 | *polA* | Yes | YPO0017 | *polA* | YPO0017 | *polA* |
| y3823 | NR | No |  |  |  |  |
| y3832 | NR | No | YPO03996 | *yhjK* | YPO3996 | *yhjK* |
| y3833 | NR | Yes |  |  |  |  |
| y3836 | *dctA* | Yes |  |  |  |  |
| y3846 | NR | No |  |  |  |  |
| y3847 | NR | No |  |  |  |  |
| y3848 | NR | No |  |  |  |  |
| y3852 | *gor* | Yes |  |  |  |  |
| y3862 | *putA* | Yes |  |  |  |  |
| y3880 | *asd* | Yes | YPO0949 | *asd* | YPO0949 | *asd* |
| y3891 | *glpD* | Yes |  |  |  |  |
| y3892 | NR | No |  |  |  |  |
| y3903 | NR | No | YPO0127 |  |  |  |
| y3916 | *ompR:0* | Yes |  |  |  |  |
| y3933 | *aroK* | Yes | YPO0151 | *aroK* | YPO0151 | *aroK* |
| y3934 | *aroB* | Yes |  |  |  |  |
| y3937 | *dam* | Yes | YPO0154 | *dam* | YPO0154 | *dam* |
| y3938 | *rpe* | Yes | YPO0155 | *rpe* | YPO0155 | *rpe* |
| y3940 | *trpS* | Yes | YPO0157 | *trpS* | YPO0157 | *trpS* |
| y3956 | *crp* | Yes | YPO0175 | *crp* | YPO0175 | *crp* |
| y3979 | NR | Yes | YPO0197 | NR | YPO0197 | NR |
| y3980 | NR | Yes | YPO0198 | NR | YPO0198 | NR |
| y3981 | NR | No | YPO0199 | NR | YPO0199 | NR |
| y3982 | NR | No |  |  |  |  |
| y3983 | *rpsL* | Yes | YPO0200 | *rpsL* | YPO0200 | *rpsL* |
| y3984 | *rpsG* | Yes | YPO0201 | *rpsG* | YPO0201 | *rpsG* |
| y3985 | *fusA* | Yes | YPO0202 | *fusA* | YPO0202 | *fusA* |
| y3986 | *tuf:6* | Yes | YPO0203 | *tufA* | TPO0203 | *tufA* |
| y3989 | *rpsJ* | Yes | YPO0209 | *rpsJ* | YPO0209 | *rpsJ* |
| y3990 | *rplC* | Yes | YPO0210 | *rplC* | YPO0210 | *rplC* |
| y3991 | *rplD* | Yes | YPO0211 | *rplD* | YPO0211 | *rplD* |
| y3992 | *rplW* | Yes | YPO0212 | *rplW* | YPO0212 | *rplW* |
| y3993 | *rplB* | Yes | YPO0213 | *rplB* | YPO0213 | *rplB* |
| y5050 | NR | Yes | YPO0214 | *rpsS* | YPO0214 | *rpsS* |
| y3994 | *rplV* | Yes | YPO0215 | *rplV* | YPO2015 | *rplV* |
| y3995 | *rpsC* | Yes | YPO0216 | *rpsC* | YPO0216 | *rpsC* |
| y3996 | *rplP* | Yes | YPO0217 | *rplP* | YPO0217 | *rplP* |
| y3998 | *rpmC* | Yes | YPO0218 | *rpmC* | YPO0218 | *rpmC* |
| y3999 | *rplN* | Yes | YPO0220 | *rplN* | YPO0220 | *rplN* |
| y4000 | *rplX* | Yes | YPO0221 | *rplX* | YPO0221 | *rplX* |
| y4001 | *rplE* | Yes | YPO0222 | *rplE* | YPO0222 | *rplE* |
| y4002 | *rpsN* | Yes | YPO0222a | *rpsN* | YPO0222a | *rpsN* |
| y4003 | *rpsH* | Yes | YPO0223 | *rpsH* | YPO0223 | *rpsH* |
| y4004 | *rplF* | Yes | YPO0224 | *rplF* | YPO0224 | *rplF* |
| y4006 | *rplR* | Yes | YPO0225 | *rplR* | YPO0225 | *rplR* |
| y4007 | *rpsE* | Yes | YPO0226 | *rpsE* | YPO0226 | *rpsE* |
| y4008 | *rpmD* | Yes | YPO0227 | *rpmD* | YPO0227 | *rpmD* |
| y4009 | *rplO* | Yes | YPO0228 | *rplO* | YPO0228 | *rplO* |
| y4010 | *secY* | Yes | YPO0229 | *secY* | YPO0229 | *secY* |
| y4011 | NR | Yes | YPO0230 | *rpmJ* | YPO0230 | *rpmJ* |
| y4012 | *rpsM* | Yes | YPO0231 | *rpsM* | YPO0231 | *rpsM* |
| y4013 | *rpsK* | Yes | YPO0232 | *rpsK* | YPO0232 | *rpsK* |
| y4014 | *rpsD* | Yes | YPO0233 | *rpsD* | YPO0233 | *rpsD* |
| y4015 | *rpoA* | Yes | YPO0234 | *rpoA* | YPO0234 | *rpoA* |
| y4016 | *rplQ* | Yes | YPO0235 | *rplQ* | YPO0235 | *rplQ* |
| y4020 | *trkA* | Yes |  |  | YPO0239 | *trkA* |
| y4022 | *fmt* | Yes | YPO0241 | *fmt* | YPO0241 | *fmt* |
| y4023 | *def* | Yes | YPO0242 | *def* | YPO0242 | *def* |
| y4027 | NR | Yes | YPO0245a | NR |  |  |
| y4028 | *aroE* | Yes |  |  |  |  |
| y4040 | *apt* | Yes | YPO3123 | *apt* | YPO3123 | *apt* |
| y4051 | NR | No |  |  |  |  |
| y4060 | NR | Yes |  |  |  |  |
| y4080 | *sodA* | Yes |  |  |  |  |
| y4089 | *glyS* | Yes | YPO4071 | *glyS* | YPO4071 | *glyS* |
| y4090 | *glyQ* | Yes | YPO4072 | *glyQ* | YPO4072 | *glyQ* |
| y4098 | NR | No |  |  |  |  |
| y4110 | *gyrB* | Yes | YPO4094 | *acrB* | YPO4094 | *acrB* |
| y4112 | *dnaN* | Yes | YPO4096 | *dnaN* | YPO4096 | *dnaN* |
| y4113 | *dnaA* | Yes | YPO4097 | *dnaA* | YPO4097 | *dnaA* |
| y4114 | NR | Yes | YPO4100 | *rimA* | YPO4100 | *rimA* |
| y4115 | *rnpA* | Yes | YPOO4101 | *rnpA* | YPO4101 | *rnpA* |
| y4117 | NR | Yes | YPO4102 | *yidC* | YPO4102 | *yidC* |
| y4118 | *trmE* | Yes |  |  |  |  |
| y4119 | NR | Yes | YPO4104 | NR |  |  |
| y4120 | NR | No |  |  |  |  |
| y4127 | *phoU* | Yes |  |  |  |  |
| y4132 | *glmS* | Yes | YPO4118 | *glmS* | YPO4118 | *glmS* |
| y4133 | *glmU* | Yes | YPO4119 | *glmU* | YPO4119 | *glmU* |
| y4134 | *atpC* | Yes | YPO4120 | *atpC* | YPO4120 | *atpC* |
| y4135 | *atpD* | Yes | YPO4121 | *atpD* | YPO4121 | *atpD* |
| y4136 | *atpG* | Yes | YPO4122 | *atpG* | YPO4122 | *atpG* |
| y4137 | *atpA* | Yes | YPO4123 | *atpA* | YPO4123 | *atpA* |
| y4138 | *atpH* | Yes | YPO4124 | *atpH* | YPO4124 | *atpH* |
| y4139 | *atpF* | Yes | YPO4125 | *atpF* | YPO4125 | *atpF* |
| y4140 | *atpE* | Yes | YPO4126 | *atpE* | YPO4126 | *atpE* |
| y4141 | *atpB* | Yes | YPO4127 | *atpB* | YPO4127 | *atpB* |
| y4142 | *atpI* | No |  |  |  |  |
| y4144 | *gidA* | Yes |  |  |  |  |

NR = not reported
