# Supplementary figures and images for: An integrated computational-experimental approach reveals Yersinia pestis genes essential across a narrow or a broad range of environmental conditions
Source: BMC Microbiol. 2017 Jul 21;17:163. doi: 10.1186/s12866-017-1073-8 (PMC5521123; doi:10.1186/s12866-017-1073-8)

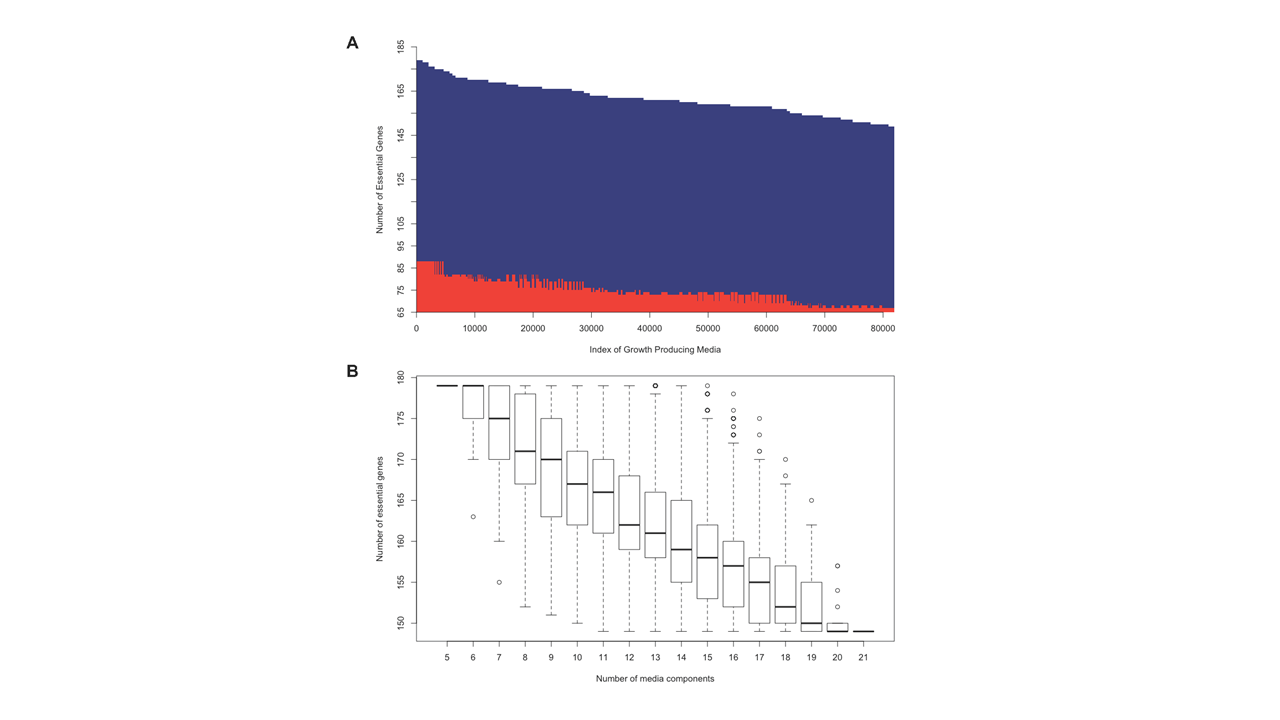

Supplement: Supplementary file 4 — Analysis of gene essentiality using the genome-scale model and 2 million randomly generated media. A. Number of essential genes (blue bars; y-axis) predicted by the model using a specific random media composition derived from the available exchange reactions in the model (x-axis). The overlap with experimentally identified genes in BAB broth media is shown as well (red bars). B. Number of essential genes (y-axis) predicted by the model using a specific media composition that is containing a given number of components of the available exchange reactions in the model (as shown in the x-axis). (TIFF 156 kb) [file 12866_2017_1073_MOESM4_ESM.tif]

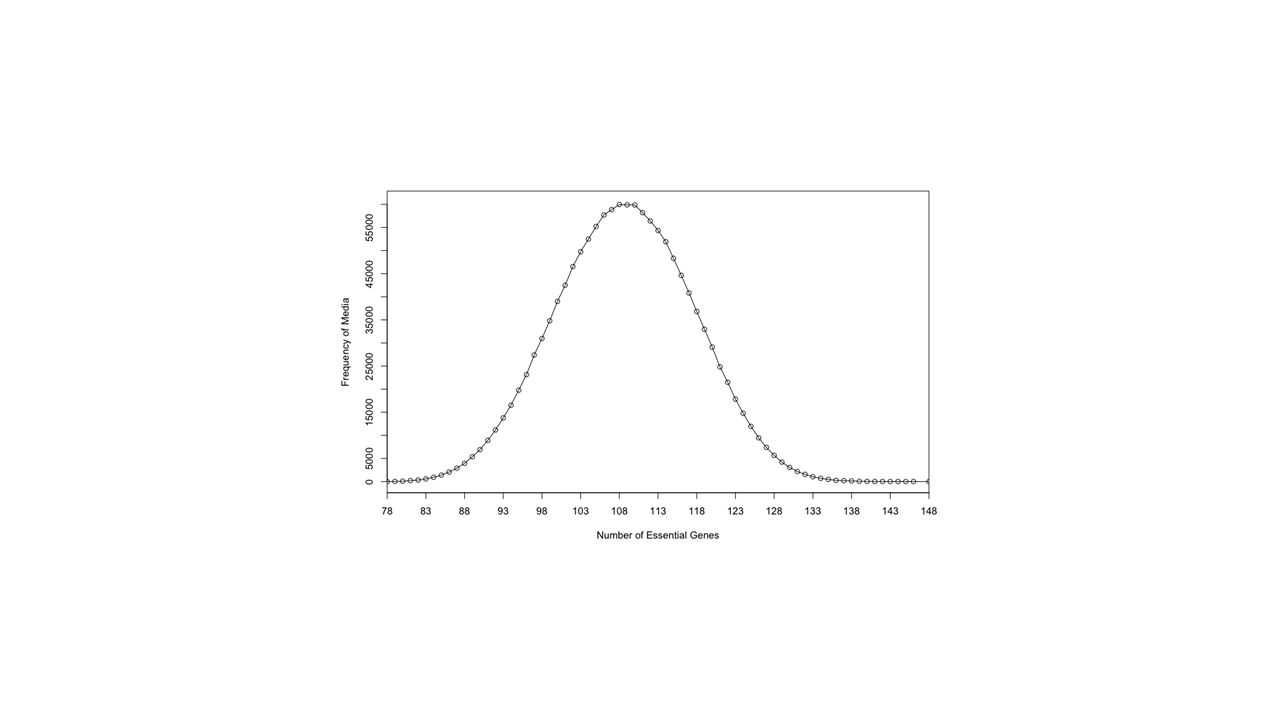

Supplement: Supplementary file 5 — Analysis of gene essentiality using the genome-scale model and 2 million randomly generated media (using available exchange reactions in the model). The x-axis list the number of genes identified as essential in a set of media (y-axis). (TIFF 95 kb) [file 12866_2017_1073_MOESM5_ESM.tif]
