# Supplementary material for: An integrated computational-experimental approach reveals Yersinia pestis genes essential across a narrow or a broad range of environmental conditions
Source: BMC Microbiol. 2017 Jul 21;17:163. doi: 10.1186/s12866-017-1073-8 (PMC5521123; doi:10.1186/s12866-017-1073-8)
Supplement: Supplementary file 6 — Overlap between experimentally identified and computationally predicted essential genes. Lines 1–54 details genes that are predicted to be essential in all computationally tested media (100% essentiality score) and are also experimentally identified to be an essential gene under the BAB broth medium. Lines 55–78 details genes that are predicted to be essential in all computationally tested media (100% essentiality score) but not experimentally identified to be an essential gene under the BAB broth medium. The gene information was collected from http://www.genome.jp on October 18 2016. (DOCX 17 kb) [file 12866_2017_1073_MOESM6_ESM.docx]

**Table S4. Overlap between experimentally identified and computationally predicted essential genes.** Lines 1-54 details genes that are predicted to be essential in all computationally tested media (100% essentiality score) and are also experimentally identified to be an essential gene under the BAB broth medium. Lines 55-78 details genes that are predicted to be essential in all computationally tested media (100% essentiality score) but not experimentally identified to be an essential gene under the BAB broth medium. The gene information was collected from http://www.genome.jp on October 18 2016.

| **No.** | **Gene ID** | **Gene Name** | **Function** |
| --- | --- | --- | --- |
| 1 | YPO1599 | *fabG* | 3-ketoacyl-ACP reductase (EC:1.1.1.100) |
| 2 | YPO3384 | *Mtn* | 5'-methylthioadenosine/S-adenosylhomocysteine nucleosidase (EC:3.2.2.9) |
| 3 | YPO1600 | *acpP* | acyl carrier protein |
| 4 | YPO2768 | *accD* | acetyl-CoA carboxylase subunit beta (EC:6.4.1.2) |
| 5 | YPO3658 | *accC* | acetyl-CoA carboxylase biotin carboxylase subunit (EC:6.4.1.2) |
| 6 | YPO1060 | *accA* | acetyl-CoA carboxylase carboxyltransferase subunit alpha (EC:6.4.1.2) |
| 7 | YPO3659 | *accB* | acetyl-CoA carboxylase biotin carboxyl carrier protein subunit (EC:6.4.1.2) |
| 8 | YPO3118 | *Adk* | adenylate kinase (EC:2.7.4.3) |
| 9 | YPO0058 | *rfaD* | ADP-L-glycero-D-manno-heptose-6-epimerase (EC:5.1.3.20) |
| 10 | YPO0672 | *parF* | acyltransferase (EC:2.3.1.51) |
| 11 | YPO3183 | *ribD* | bifunctional diaminohydroxyphosphoribosylaminopyrimidine deaminase/5-amino-6-(5-phosphoribosylamino)uracil reductase (EC:1.1.1.193 3.5.4.26) |
| 12 | YPO1395 | *msbA* | lipid transporter ATP-binding protein/permease |
| 13 | YPO0658 | *Rib* | 3,4-dihydroxy-2-butanone 4-phosphate synthase |
| 14 | YPO0040 | *Gmk* | guanylate kinase (EC:2.7.4.8) |
| 15 | YPO0486 | *folA* | dihydrofolate reductase (EC:1.5.1.3) |
| 16 | YPO2769 | *folC* | bifunctional folylpolyglutamate synthase/dihydrofolate synthase (EC:6.3.2.17) |
| 17 | YPO0648 | *folB* | bifunctional dihydroneopterin aldolase/dihydroneopterin triphosphate 2'-epimerase (EC:4.1.2.25) |
| 18 | YPO3501 | *folP* | dihydropteroate synthase (EC:2.5.1.15) |
| 19 | YPO3430 | *coaE* | dephospho-CoA kinase (EC:2.7.1.24) |
| 20 | YPO1605 | *Tmk* | thymidylate kinase (EC:2.7.4.9) |
| 21 | YPO0474 | *ribF* | bifunctional riboflavin kinase/FMN adenylyltransferase (EC:2.7.1.26 2.7.7.2) |
| 22 | YPO4119 | *glmU* | bifunctional N-acetylglucosamine-1-phosphate uridyltransferase/glucosamine-1-phosphate acetyltransferase (EC:2.7.7.23) |
| 23 | YPO0312 | *plsB* | glycerol-3-phosphate acyltransferase (EC:2.3.1.15) |
| 24 | YPO0654 | *rfaE* | bifunctional heptose 7-phosphate kinase/heptose 1-phosphate adenyltransferase (EC:2.7.-.-) |
| 25 | YPO1505 | *folE* | GTP cyclohydrolase I (EC:3.5.4.16) |
| 26 | YPO2222 | *ribA* | GTP cyclohydrolase II (EC:3.5.4.25) |
| 27 | YPO3407 | *yadF* | carbonic anhydrase |
| 28 | YPO3400 | *folK* | 2-amino-4-hydroxy-6-hydroxymethyldihydropteridine pyrophosphokinase (EC:2.7.6.3) |
| 29 | YPO1400 | *kdsB* | 3-deoxy-manno-octulosonate cytidylyltransferase (EC:2.7.7.38) |
| 30 | YPO2021 | *kdsA* | 2-dehydro-3-deoxyphosphooctonate aldolase (EC:2.5.1.55) |
| 31 | YPO1057 | *lpxB* | lipid-A-disaccharide synthase (EC:2.4.1.182) |
| 32 | YPO1598 | *fabD* | malonyl CoA-ACP transacylase (EC:2.3.1.39) |
| 33 | YPO0931 | *metK* | S-adenosylmethionine synthetase (EC:2.5.1.6) |
| 34 | YPO0055 | *kdtA* | 3-deoxy-D-manno-octulosonic-acid transferase |
| 35 | YPO1106 | *ppnK* | inorganic polyphosphate/ATP-NAD kinase (EC:2.7.1.23) |
| 36 | YPO3500 | *glmM* | phosphoglucosamine mutase (EC:5.4.2.10) |
| 37 | YPO1867 | *pgsA* | phosphatidylglycerophosphate synthetase (EC:2.7.8.5) |
| 38 | YPO3758 | *coaA* | pantothenate kinase (EC:2.7.1.33) |
| 39 | YPO0048 | *Dfp* | bifunctional phosphopantothenoylcysteine decarboxylase/phosphopantothenate synthase (EC:4.1.1.36 6.3.2.5) |
| 40 | YPO0364 | *Psd* | phosphatidylserine decarboxylase (EC:4.1.1.65) |
| 41 | YPO3273 | *pssA* | phosphatidylserine synthase (EC:2.7.8.8) |
| 42 | YPO0053 | *coaD* | phosphopantetheine adenylyltransferase (EC:2.7.7.3) |
| 43 | YPO2391 | *ribE* | riboflavin synthase subunit alpha (EC:2.5.1.9) |
| 44 | YPO3182 | *ribH* | 6,7-dimethyl-8-ribityllumazine synthase (EC:2.5.1.9) |
| 45 | YPO1396 | *lpxK* | tetraacyldisaccharide 4'-kinase (EC:2.7.1.130) |
| 46 | YPO0926 | *tktA* | transketolase (EC:2.2.1.1) |
| 47 | YPO1054 | *lpxD* | UDP-3-O-[3-hydroxymyristoyl] glucosamine N-acyltransferase (EC:2.3.1.-) |
| 48 | YPO1056 | *lpxA* | UDP-N-acetylglucosamine acyltransferase (EC:2.3.1.129) |
| 49 | YPO0561 | *lpxC* | UDP-3-O-[3-hydroxymyristoyl] N-acetylglucosamine deacetylase (EC:3.5.1.-) |
| 50 | YPO3075 | NR | UDP-2,3-diacylglucosamine hydrolase |
| 51 | YPO3243 | *gmhA* | phosphoheptose isomerase (EC:5.-.-.-) |
| 52 | YPO3577 | NR | D-arabinose 5-phosphate isomerase |
| 53 | YPO0056 | *rfaC* | ADP-heptose:LPS heptosyl transferase I |
| 54 | YPO0057 | *rfaF* | ADP-heptose:LPS heptosyltransferase II |
| 55 | YPO1222 | *ompC* | porin |
| 56 | YPO1603 | *pabC* | 4-amino-4-deoxychorismate lyase (EC:4.1.3.38) |
| 57 | YPO1773 | *pabB* | para-aminobenzoate synthase component I (EC:4.1.3.-) |
| 58 | YPO0169 | *pabA* | para-aminobenzoate synthase component II (EC:2.6.1.85) |
| 59 | YPO3412 | *speD* | S-adenosylmethionine decarboxylase (EC:4.1.1.50) |
| 60 | YPO2751 | *aroC* | chorismate synthase (EC:4.2.3.5) |
| 61 | YPO2188 | *Cls* | cardiolipin synthetase (EC:2.7.8.-) |
| 62 | YPO3939 | *glgA* | glycogen synthase (EC:2.4.1.21) |
| 63 | YPO3940 | *glgC* | glucose-1-phosphate adenylyltransferase (EC:2.7.7.27) |
| 64 | YPO1074 | NR | D,D-heptose 1,7-bisphosphate phosphatase |
| 65 | YPO3578 | NR | 3-deoxy-D-manno-octulosonate 8-phosphate phosphatase (EC:3.1.3.45) |
| 66 | YPO0322 | *tyrB* | aromatic amino acid aminotransferase (EC:2.6.1.57) |
| 67 | YPO3726 | *aceB* | malate synthase (EC:2.3.3.9) |
| 68 | YPO0117 | *metF* | 5,10-methylenetetrahydrofolate reductase (EC:1.5.1.20) |
| 69 | YPO1390 | *aroA* | 3-phosphoshikimate 1-carboxyvinyltransferase (EC:2.5.1.19) |
| 70 | YPO3215 | *aroL* | shikimate kinase II (EC:2.7.1.71) |
| 71 | YPO3411 | *speE* | spermidine synthase (EC:2.5.1.16) |
| 72 | YPO1540 | *galF* | UTP-glucose-1-phosphate uridylyltransferase (EC:2.7.7.9) |
| 73 | YPO1539 | *galU* | UTP-glucose-1-phosphate uridylyltransferase (EC:2.7.7.9) |
| 74 | YPO2298 | *phoA* | alkaline phosphatase (EC:3.1.3.1) |
| 75 | YPO2063 | *msbB* | lipid A biosynthesis (KDO)2-(lauroyl)-lipid IVA acyltransferase (EC:2.3.1.-) |
| 76 | YPO1650 | NR | hypothetical protein |
| 77 | YPO3632 | *ddg* | lipid A biosynthesis palmitoleoyl acyltransferase |
| 78 | YPO0416 | NR | lipopolysaccharide core biosynthesis protein |

NR = not reported
